# Nurturing compassion in schools: A randomized controlled trial of the effectiveness of a Compassionate Mind Training program for teachers

**DOI:** 10.1371/journal.pone.0263480

**Published:** 2022-03-01

**Authors:** Marcela Matos, Isabel Albuquerque, Ana Galhardo, Marina Cunha, Margarida Pedroso Lima, Lara Palmeira, Nicola Petrocchi, Kirsten McEwan, Frances A. Maratos, Paul Gilbert

**Affiliations:** 1 University of Coimbra, Faculty of Psychology and Educational Sciences, Center for Research in Neuropsychology and Cognitive and Behavioural Intervention (CINEICC), Coimbra, Portugal; 2 Instituto Superior Miguel Torga, Coimbra, Portugal; 3 Universidade Portucalense, Infante D. Henrique, Porto, Portugal; 4 John Cabot University, Rome, Italy; 5 University of Derby, College of Health, Psychology & Social Care, Derby, United Kingdom; Prince Sattam Bin Abdulaziz University, College of Applied Medical Sciences, SAUDI ARABIA

## Abstract

**Objectives:**

Schools are experiencing an unprecedented mental health crisis, with teachers reporting high levels of stress and burnout, which has adverse consequences to their mental and physical health. Addressing mental and physical health problems and promoting wellbeing in educational settings is thus a global priority. This study investigated the feasibility and effectiveness of an 8-week Compassionate Mind Training program for Teachers (CMT-T) on indicators of psychological and physiological wellbeing.

**Methods:**

A pragmatic randomized controlled study with a stepped-wedge design was conducted in a sample of 155 public school teachers, who were randomized to CMT-T (*n* = 80) or a waitlist control group (WLC; *n* = 75). Participants completed self-report measures of psychological distress, burnout, overall and professional wellbeing, compassion and self-criticism at baseline, post-intervention, and 3-months follow-up. In a sub-sample (CMT-T, *n* = 51; WLC *n* = 36) resting heart-rate variability (HRV) was measured at baseline and post-intervention.

**Results:**

CMT-T was feasible and effective. Compared to the WLC, the CMT-T group showed improvements in self-compassion, compassion to others, positive affect, and HRV as well as reductions in fears of compassion, anxiety and depression. WLC participants who received CMT-T revealed additional improvements in compassion for others and from others, and satisfaction with professional life, along with decreases in burnout and stress. Teachers scoring higher in self-criticism at baseline revealed greater improvements post CMT-T. At 3-month follow-up improvements were retained.

**Conclusions:**

CMT-T shows promise as a compassion-focused intervention for enhancing compassion, wellbeing and reducing psychophysiological distress in teachers, contributing to nurturing compassionate, prosocial and resilient educational environments. Given its favourable and sustainable effects on wellbeing and psychophysiological distress, and low cost to deliver, broader implementation and dissemination of CMT-T is encouraged.

## Introduction

The promotion of mental wellbeing constitutes a public health priority, with mental health difficulties being leading causes of disability and representing a long-lasting and major economic, social and health burden [1]. The United Nations 2030 Agenda for Sustainable Development [2] highlights the importance of promoting health and wellbeing for all ages and accentuates the need to foster compassion and empathy to tackle global inequality and cultivate peaceful and resilient societies.

Schools are withstanding an unprecedented mental health crisis and have become increasingly stressful environments for the whole educational community [3]. Facing the multiple challenges of working in schools (e.g., excessive workload, time pressures, bureaucracy, pupil disruptive behaviours), teachers report high levels of stress and burnout within all education sectors and across countries [4]. In Portugal, the latest education sector research revealed that 75% of teachers present high levels of burnout, with 25% reporting extreme burnout and 84% intending to leave the profession due to stress and competitive pressures [5]. Along with this retention crisis in the teaching profession, long-term teacher stress is associated with a range of poor wellbeing and professional outcomes, which carry significant socioeconomic costs. For example, absenteeism and staff turnover, reduced self-efficacy [6, 7], poor wellbeing and burnout [8–10], with adverse consequences to mental [6, 11] and physical health [5, 12, 13]. The prolonged activation of stress-responsive physiological systems (e.g., hypothalamic-pituitary-adrenal axis; sympathetic nervous system) not only impairs psychological wellbeing [14] but also negatively affects neuroendocrine (e.g., cortisol), autonomic (e.g., heart rate variability, HRV) and immune-inflammatory responses [14, 15], with long-lasting changes in stress-related gene expression [16, 17], which have a detrimental impact on mental and physical health.

In addition, teacher’s stress negatively impacts pupils’ social adjustment, academic performance [18] and mental wellbeing [19–21]. For example, Oberle and Schonert-Reichl [22] revealed that student’s cortisol levels were much higher in classrooms led by a teacher who reported feeling overwhelmed. Longitudinal studies have further revealed that teachers reporting higher burnout early in the year have classrooms presenting more behavioural problems across the year [23]. When teachers report lower levels of work-related stress, students find those teachers more interested and enthusiastic in teaching [24], which influences pupils’ motivation and affect [25, 26]. In addition, teachers’ wellbeing is linked to an array of positive outcomes, such as positive classroom processes (e.g., teachers’ active support towards students, classroom social climate), as well as students’ self-efficacy, subjective wellbeing, achievement, and motivation and attitude towards learning [27, 28].

One source of teacher stress is the competitive dynamic of modern neoliberal societies that have come to texture learning environment and schools [29, 30]. This competitiveness is a major source of stress, which is particularly evident in schools. This competitiveness is a major source of stress, affecting both teachers (e.g., heavier workloads, achievement focus, performance evaluation) and pupils (e.g., focus on academic achievement, self-interest). In fact, self- and other-focused competitive pressures have been highlighted as a key source for teachers and pupil mental health problems [31–34], and underlie fears of failure, feelings of shame and negative social comparisons, self-criticism and resistances to compassion [35–37].

Schools are crucial social arenas that can promote competitiveness and self-interest or cultivate prosociality and compassion, and where these two distinct motivational systems: competitiveness vs compassion, with their social information flows and biological patterns, become choreographed and played out [36, 38]. Contrary to competitive motives, when individuals are caring and sharing, particular physiological systems linked to affiliation and social connectedness are stimulated [e.g., parasympathetic nervous system, oxytocinergic system), which contribute to wellbeing, stress management and emotion regulation, 39, 40]. In fact, studies show that social relationships and adversity impact one’s physiology even at the epigenetic level [e.g., differential methylation in oxytocin receptor gene_OXTR, 17].

Compassion, commonly defined as a sensitivity to suffering in self and others with a commitment to try to alleviate and prevent it [41, 42], is an innate prosocial motivation that evolved with the mammalian caring system. Compassion has benefits for mental health, emotion regulation and social relationships [e.g., 43–47]. Furthermore, compassion positively impacts physiological health, including influences in genetic expression [e.g., lower levels of CTRA-related gene expression, 48; epigenetic profiles of the OXTR, 49]. Experimental studies have also documented that cultivating compassion impacts physiological systems, reducing arousal and increasing parasympathetic activation [e.g., heart-rate variability, HRV, 50–53], decreasing sensitivity to threat [49], and activating specific neural circuits distinct from empathy and mindfulness training [54].

Given the burgeoning evidence of the numerous benefits of compassion over the past decade, several interventions have been developed that specifically aim to cultivate compassion [51]. A growing body of empirical evidence has testified their positive impact on mental and physical wellbeing and prosocial behaviour [55–57]. One of these approaches is an evolutionary and biopsychosocial evidence-based approach called Compassionate Mind Training [CMT; 42, 58, 59]. CMT was developed as an intervention for the general public and comprises psychoeducation and a set of core compassion and mindfulness practices taken from Compassion Focused Therapy [CFT; 58, 60, 61]. CFT, based upon evolutionary psychology, attachment theory, psychological science, and an understanding of motivational systems, is a transdiagnostic therapy for individuals dealing with shame and self-criticism, and currently delivered to patients with a wide range of mental and physical health difficulties. CFT has been shown to be an effective approach in a multitude of clinical conditions and symptoms [62, 63].

CMT is designed to activate and develop evolved, affiliative care-focused motivational systems and emotions in order to down-regulate competitive and threat-focused systems and stimulate psychological and neurophysiological processes (primarily associated with the parasympathetic system) conductive to better emotion regulation, wellbeing, health and social relationships [58, 60, 61]. CMT uses a range of evidence-based practises such as breathing techniques, visualisations and behavioural practises to stimulate the vagus nerve, balance the autonomic nervous system [64] and recruit various neuro circuits associated with compassion [53, 54]. CMT strives to cultivate a compassionate mind which includes the three interactive flows of compassion: the ability to be compassionate toward the self, and others, as well as to receive compassion from others. Each of these make a contribution to well-being and prosocial behaviour, but each can also have fears, blocks, and resistances that need to be tackled [58, 61, 65]. CMT also addresses key issues linked to competitive threats such as self-criticism and fears of compassion [58, 60, 65].

CMT seeks to develop mental competencies and physiological states that promote two fundamental interrelated processes of compassion. The first is the ability to be sensitive and turn towards suffering in self and others, to tolerate and engage with this suffering, rather than avoid it or dissociate, which is related to many attributes, such as the motivation to care and the capacity for feeling sympathy and empathy. The second is the commitment to alleviate and prevent suffering and requires a particular set of affiliative skills in the sphere of attention, emotion, cognition and behaviour conducive to the development of a compassionate mind [58, 61]. Importantly, this means that individuals need to develop the courage to move towards suffering and potentially painful situations or mental states, but also the wisdom of knowing what to do. Hence CMT is centred around the development of competencies needed to *courageously turn towards and engage* with difficulties in self and others, and a variety of skills linked to reasoning, mentalizing and emotional regulation, which enable compassion motives to be translated into compassionate actions [42, 61, 65].

CMT has been tailored for use with different formats (e.g., length, practices) in nonclinical populations, namely the general public [66, 67], health care educators and providers [68], mental health professionals [69], nurses [70, 71], firefighters [72] and psychotherapy students [73]. There is a mounting evidence base for CMT effectiveness in improving mental and physiological health and prosocial behaviour [56, 59, 62, 63]. A pilot randomised controlled study in a community sample [67, 74] found that a brief CMT intervention promoted beneficial psychological changes associated with wellbeing and improvements in HRV. Higher HRV is indicative of higher parasympathetic nervous system outflow via the vagus nerve activity and is associated with self-compassion, feelings of perceived safeness and warmth and greater ability to self-soothe when stressed [e.g., 64, 75–78]. A recent uncontrolled study demonstrated the promising effects of an 8-week CMT group intervention for the general public, in increasing levels of compassion, positive emotions and wellbeing, and reducing self-criticism and psychological distress, and validated the maintenance of these changes at 3-month follow-up [66]. In addition, a randomised controlled trial of CFT intervention as guided self-help in an adult community sample reported positive effects on wellbeing and psychopathological indicators up to 12 months after baseline [79].

This knowledge that promoting compassion (for self and others) has a range of psychological and physiological benefits for mental and physical wellbeing and prosocial behaviour [55] needs to be prioritised and incorporated into teacher education and into schools [36, 80] and is aligned with international guidelines for the promotion of health and wellbeing [2, 27]. Likewise, in Portugal, government recommendations for Education emphasise the importance of advancing health literacy and social-emotional competencies in educational settings to promote health and psychological wellbeing [81, 82].

Recognising the growing problems of stress in schools and the crisis within the teaching profession, there are now a number of projects to improve wellbeing and resilience in teachers, with those stemming from compassion and mindfulness-based interventions demonstrating to be particularly effective [83–86]. One of these is the Compassionate Schools Research Initiative, which implemented and evaluated a 6-module Compassion Mind Training for Teachers (CMT-T), building upon previous CFT and CMT programs [67], in schools in Portugal and the UK. In the UK, an earlier version of the CMT-T, applied in 70 teachers and support staff, was feasible and well-received, with participants (*N* = 34) positively evaluating the curriculum and the practices and its helpfulness for dealing with emotional difficulties [32]. A subsample of 20 teachers completed pre- and post-self-report assessments and showed significant decreases in self-criticism and increases in self-compassion at post-intervention; however, burnout and psychopathology did not significantly change [32].

In Portugal, an uncontrolled pilot study with 31 teachers showed that CMT-T was feasible and well-received, increased teachers’ compassion for others, self-compassion and compassion to others motivations and actions, and reduced depression, stress and fears of compassion to others [87]. Importantly, when self-criticism was controlled for, a decrease in burnout and an increase in self-compassion and in satisfaction with professional life were additionally found. In line with previous studies [88–90], this finding emphasises the importance of targeting self-criticism across the intervention, given its key role in how CMT-T operates in promoting teachers’ abilities to be compassionate towards themselves and thus fostering their wellbeing and diminishing psychological distress. Furthermore, this study found that fears of compassion for others mediated the impact of CMT-T on teachers’ burnout and that self-compassion mediated the intervention effect on psychological wellbeing. Thus, these findings emphasise the importance of targeting both the fears, blocks and resistances to compassion alongside cultivating self-compassion abilities in teachers to decrease distress and promote psychological wellbeing. Finally, the qualitative experience of the CMT-T for both participants and facilitators suggested that future iterations of the program might benefit from extending the duration of sessions and length of the program [87]. Another study explored the international utility of the CMT-T and concluded this is a feasible, useful and effective intervention in cross-cultural educational settings, not only in terms of introducing and promoting a compassion-based school ethos but also on cultivating the psychological wellbeing of those working in education [91].

Therefore, CMT-T may provide a suitable approach to counteract the current challenges in educational settings and inspire a shift from competitiveness/threat-based to compassionate/affiliative motivational systems, to improve educators’ stress regulation, prosocial qualities, behaviour and wellbeing. Still, a further evaluation of the pragmatic effectiveness of a refined version of CMT-T on teachers’ wellbeing and mental health, using a larger sample size and a randomized controlled design, is needed. Moreover, the impact of the CMT-T on affiliative- and stress-related biophysiological markers, particularly on the parasympathetic activity as measured through HRV [40, 56], warrants empirical support. Additionally, as an alternative to traditional parallel designs, the use of a stepped wedge design would allow exposing both the intervention and control groups to the CMT-T, while also establishing a within-subjects psychological and physiological baseline in the control group and controlling for the confounding effect of time [92].

Given the growing research on the multidimensional benefits of compassion cultivation, and pilot studies on CMT-T, the current study sought to further explore the feasibility and effectiveness of the CMT-T on teachers’ psychological distress, wellbeing and compassion to self and others, by evaluating a refined 8-week version of the CMT-T and using a randomised controlled and stepped wedge design. CMT-T specifically aimed at promoting positive affect and satisfaction with professional life and reducing symptoms of depression, anxiety, stress, and burnout (primary outcome variables), by increasing the flows of compassion (for self, for others and from others), self-compassion and compassion to others motivations and actions, and by diminishing fears of compassion (for self, for others and from others) and self-criticism (secondary/process outcome variables). Furthermore, the present study aimed to explore the impact of the CMT-T on heart rate variability (HRV), an indicator of vagal regulatory activity and a physiological marker of a person’s ability to flexibly respond to environmental challenges and regulate emotional responses [56, 75], which has been proposed as a primary measure to assess and train compassion [56]. It was hypothesised that CMT-T produces significant increases in HRV. In addition, the present study aimed at examining the impact of the CMT-T on teachers who received the intervention after a period of baseline observations where they acted as controls. It was hypothesised that these participants would reveal no significant changes from baseline to pre-intervention but would reveal significant changes in both outcome and process variables after receiving the CMT-T intervention.

In light of previous research pointing to the role of individual differences in self-criticism on how individuals respond to compassion-based interventions in general [88–90], and on the impact of CMT-T in particular [87], we also explored how self-criticism might influence the effects of the CMT-T intervention. Baseline self-criticism was hypothesized to impact the CMT-T effects on the process and outcome variables. Furthermore, we examined whether changes from pre-to-post CMT-T were different when comparing high and low self-critics, as well as whether there were differences between these two groups in the magnitude of change. In addition, the current study aimed to examine whether the effects of attending the CMT-T were sustained at 3-month post-intervention.

Finally, given that the inter-relationship between the three flows of compassion (i.e., compassion for others, being open to compassion from others, and self-compassion) is a key aspect of the CFT/CMT approach [61, 93], we explored whether the associations between the flows of compassion would change from before to after the CMT-T, particularly whether these were strengthened after training.

## Methods

### Study design

The current study was a pragmatic two-arm randomized controlled trial (RCT), with one intervention group (CMT-T) and one waitlist control group (WLC), and a stepped-wedge design where all groups and participants in groups were offered the intervention. This study was approved by the Ethics Committee of the Faculty of Psychology and Educational Sciences of the University of Coimbra (CEDI22.03.2018), and registered at ClinicalTrials.gov (Identifier: NCT05107323; Compassionate Schools: Feasibility and Effectiveness Study of a Compassionate Mind Training Program to Promote Teachers Wellbeing). The findings of this RCT are reported conform the Consolidated Standards of Reporting Trials (CONSORT) guidelines [94; see S1 File] and the Journal Article Reporting Standards (JARS) for research in psychology [95].

Given the stepped wedge design, there were four assessment moments in the study: 1) Time 1 (T1)—baseline/pre-intervention assessment, was completed by the CMT-T group and the WLC group during the week previous to the start of the CMT-T intervention; 2) Time 2 (T2)—post-intervention assessment one was completed by CMT-T group and WLC group during the first-week post-intervention; 3) Time 3 (T3)—post-intervention assessment two, was completed by WLC group participants one week after they had also received the CMT-T intervention; 4) 3-months Follow-up assessment, this was conducted three months after the CMT-T conclusion (for all participants who completed the intervention). In T1, T2, and T3 all participants completed a set of self-report questionnaires assessing primary and secondary outcomes. A subsample of participants (*n* = 55 in the experimental group, and *n* = 40 in the WLC group) underwent the HRV measurement at T1, T2, and T3. In the 3-month follow-up assessment, only self-report data were collected.

The study was implemented between October 2018 and August 2019, across the following phases: 1) October/November 2018 (T1 for CMT-T Groups 1&2 and for WLC Groups 1&2); 2) December 2018/January 2019 (T2 for CMT-T Groups 1&2 and for WLC Group 1&2; WLC Group 1&2 started the intervention; T1 for WLC Group 3); 3) March 2019 (T3 for WLC Group 1&2 after receiving the intervention; T2 for WLC Group 3, before starting the intervention; T1 for CMT-T Group 3); 4) May 2019 (T2 for CMT-T 3 Group; T3 for WLC Group 3, after receiving the intervention); 5) Follow-up assessment was conducted between March-August 2019 by all participants who received the intervention 3-months after their respective group finished the CMT-T.

### Participants and recruitment

In May/June 2018, participants were recruited amongst teaching staff in public schools (pre to high school grades) in the centre region of Portugal (Viseu and Coimbra districts). Schools’ boards were invited to participate in the study. Four schools enrolled in the project, provided further ethical approval and invited all teaching staff to attend an informative 2-h session about the study. This recruitment session was led by the research team in each school and included a brief description of the study aims, procedures, conditions for participation and ethical considerations. Additionally, a leaflet containing this information was distributed among staff by the schools’ Board. Teachers interested in participating in the study contacted the research team via email. They were then contacted via email and assessed for inclusion criteria and required to provide informed consent. Informed consent clarified the voluntary, confidential and anonymous nature of the study and data protection rights. Each participant created a unique and numerical code to guarantee confidentiality that was used in all assessment tasks. The assessment moments and intervention were conducted at the schools.

**Fig 1:**
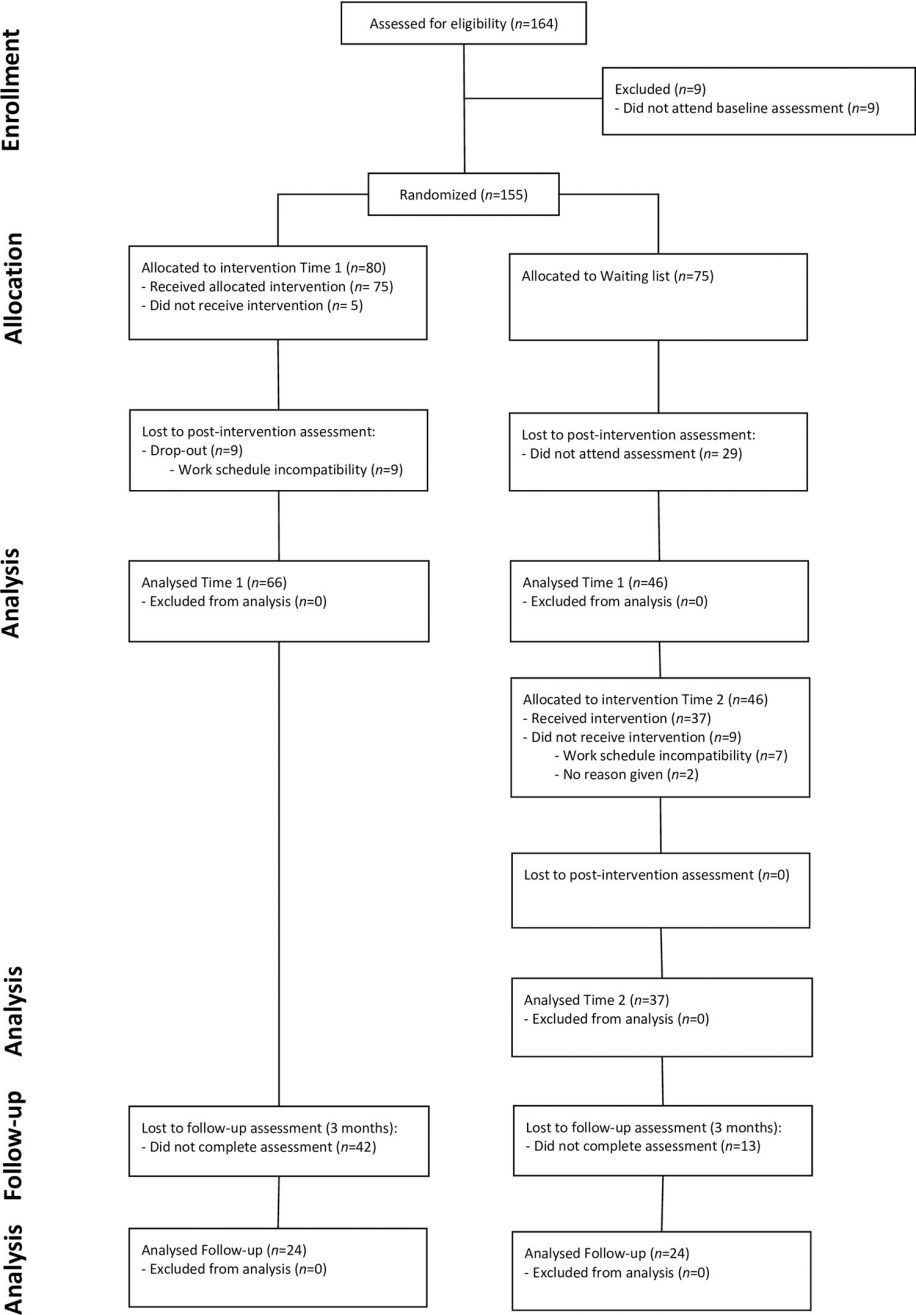
Flowchart of study participants.

Participants were eligible for participation if they: (a) were teachers in the enrolled schools; and (b) provided informed consent. Fig 1 displays the flow of participants. Overall, 164 teachers showed interest to take part in the study and met the eligibility criteria. Nine teachers failed to attend the baseline assessment. After baseline assessment, 155 participants were randomly assigned within each school to either the CMT-T intervention (CMT-T group) or the waitlist control (WLC) group. Each WLC group started the CMT-T after their parallel CMT-T group completed the intervention, i.e., after approximately two months. In total, six groups received the CMT-T (*n* per group *M* = 18), which was delivered in the school setting. From the initial 80 participants allocated to the CMT-T group, five failed to attend any session. Moreover, nine participants dropped-out (11.25%) because of work schedule incompatibility, and were excluded from further analysis. From the initial 75 participants allocated to the WLC group, 29 did not attend the second assessment and were also excluded from the analysis. The remaining 46 participants from the WLC group were allocated to the intervention at Time 2. From those, 37 received the intervention, and nine opted not to receive the intervention. There were no dropouts from the intervention at Time 2.

Sociodemographic characteristics of participants are presented om Table 1. The study sample was composed of 155 teachers working in public schools from Portugal’s centre region. Participants’ age ranged from 25 to 63 years old, with a mean age of 51.35 (*SD* = 7.24). The majority of the participants was female (92.9) and held a graduate/honors degree (67.1%) or had completed a master’s degree (23.9%). Most participants were married (65.8%), 16.8% were divorced, and 13.5% were single. In terms of teaching-related characteristics, participants had been working as teachers for 10 months to 41 years (*M* = 27.01, *SD* = 8.29), and taught in several grade levels: 12.3% were preschool teachers, 16.1% taught in elementary school, 15.4% in middle school, 36.1% in high school, and 14.8% in special education. The participants qualified as middle or high school grade level teachers taught in the following content areas: 25.5% languages, 6.5% social and human sciences, 13.5% mathematics and experimental sciences, 2.6% artistic expression, 0.4% physical education, and 3.9% information technologies. Eight teachers did not provide information about these aspects. Regarding school setting, teachers were recruited in two large middle/high schools from an urban area, in one cluster of schools that included large schools from a semi-urban area and small schools from rural areas, and in one cluster of schools from an urban area including a large school and small schools from urban as well rural areas.

**Table 1:** Baseline characteristics of the participants (*N* = 155).

Characteristic	Total (*N* = 155)	CMT-T (*n* = 80)	WLC (*n* = 75)	Test statistic (*p*-value)
Age, years				*t*_(153)_ = .32 (*p* = .747)
Mean (SD)	51.35 (7.24)	51.54 (8.21)	51.16 (6.09)	
Range	25–63	25–63	28–62	
Sex *n* (%)				χ ^2^ = .04 (*p* = .840)
Male	11 (7.1)	6 (7.5)	5 (6.7)	
Female	144 (92.9)	74 (92.5)	70 (93.3)	
Marital status *n* (%)				χ ^2^ = 2.28 (*p* = .684)
Single	21 (13.5)	13 (16.3)	8 (10.7)	
Married/Registered partnership	102 (65.8)	52 (65)	50 (66.6)	
Divorced	26 (16.8)	12 (15)	14 (18.7)	
Widowed	6 (3.9)	3 (3.8)	3 (4)	
Education level *n* (%)				χ ^2^ = 12.70 (*p* = .005)
Bachelor	3 (1.9)	-	3 (4)	
Graduate/Honors	104 (67.1)	60 (75)	44 (58.7)	
Postgraduate specialisation	11 (7.1)	1 (1.3)	10 (13.3)	
Masters	37 (23.9)	19 (23.8)	18 (24)	
Years of teaching experience, years				*t*_(153)_ = .07 (*p* = .946)
Mean (SD)	27.01 (8.29)	27.05 (9.56)	26.96 (6.73)	
Range	0.83–41	0.83–41	2–38	
Teaching grade level *n* (%)				χ ^2^ = 18.54 (*p* = .001)
Preschool	19 (12.3)	8 (10.0)	11 (14.7)	
Elementary school	25 (16.1)	18 (22.5)	7 (9.3)	
Middle school	24 (15.5)	4 (5.2)	20 (26.7)	
High school	56 (36.1)	34 (42.2)	22 (29.3)	
Special education (All levels)	23 (14.8)	11 (13.8)	12 (16.0)	
Missings	8 (5.2)	5 (6.3)	3 (4.0)	
Teaching subject areas *n* (%)				χ ^2^ = 11.26 (*p* = .128)
Several areas (Pre and Elementary School)	44 (28.4)	26 (32.5)	18 (24.0)	
Languages	38 (24.5)	13 (16.3)	25 (33.3)	
Social and human sciences	10 (6.5)	8 (10.0)	2 (2.7)	
Mathematics and experimental sciences	21 (13.5)	12 (15.0)	9 (12)	
Artistic expression	4 (2.6)	1 (1.2)	3 (4.0)	
Physical education	1(0.6)	1 (1.2)	0 (0.0)	
Information technologies	6 (3.9)	3 (3.7)	3 (4.0)	
Special Education (All areas)	23 (14.8)	11 (13.8)	12 (16.0)	
Missings	8 (5.2)	5 (6,3)	3 (4.0)	

*Note*. CMT-T = Compassionate Mind Training for Teachers; WLC = Waitlist control group.

Independent samples *t* tests and chi-squared tests indicated that the intervention group and the waitlist control group were similar in age, sex, marital status, years of teaching and teaching subject areas, indicating a successful randomization. Significant differences were found between the two groups in education level and teaching grade level.

### Intervention

#### The Compassionate Mind Training Program for Teachers (CMT-T)

The Compassionate Mind Training program for Teachers (CMT-T) is a compassion mind training intervention tailored for teaching staff and delivered in a group format across eight sessions of approximately 2.5 hours each. The 8-week CMT-T is a refined version of the 6-week CMT-T curriculum [87], which was developed based on an earlier version of the program for school staff designed and tested by Maratos et al. [32] and on a brief CMT program for the general public [67]. The feasibility and preliminary effectiveness of the pilot version of the CMT-T showed promising results in Portugal and the UK [87, 91]. However, the implementation and evaluation of this pilot version indicated that the program could be improved in a few aspects [87]. The CMT-T was hence refined to accommodate these alterations, specifically in the number of sessions, duration of the sessions, and the tackling of the self-criticism topic (cf. Results section for a detailed description of the Adaption). There are six different modules addressed during the 8-sessions of the refined CMT-T. A brief overview of the six modules is presented in Table 2. In each session, besides presenting relevant theoretical constructs addressed in that session, participants are invited to complete experiential exercises, compassion and mindfulness meditation practices, and work in small groups to share their experiences, followed by a plenary session.

**Table 2:** CMT-T modules, contents, practices and exercises.

Module	Theme	Contents	Practices and Exercises
**1**	Compassion and the evolutionary nature of the human mind	How do human minds work? What is compassion? (evolutionary model framework) Facilitators and inhibitors of compassion	*Soothing Rhythm Breathing* *Compassionate facial expression and voice tone* *Compassionate listening*
**2**	Understanding the functions of our emotions: The three circles model	The three affect systems: Threat, Drive and Safeness/Soothing What are the functions of human emotions? Functional analysis of Threat, Drive and Safeness/Soothing emotions	*Soothing Rhythm Breathing Compassionate facial expression and voice tone nerve*. *Spotlight of Attention Exercise* *Mindfulness of the Senses*
**3**	Building a compassionate mind/self	The three compassion flows The two psychologies/components of compassion Compassion competencies and skills The compassionate self–qualities How can we develop a warm and caring relationship with ourselves and with others? How to manage fears, blocks and resistances to compassion	*Safe place* *Cultivating the Ideal Compassionate Self* *Embodiment of the Compassionate self* *Directing Compassion to Others and to Self*
**4**	Working with emotions: The role of compassionate self	Functional analysis of emotions Emotional conflicts Using the compassionate self to work with difficult emotions How to manage fears, blocks and resistances to compassion	*Ideal Compassionate Other* *Multiple selves exercise* *Functional analysis of emotions*
**5**	Understanding and working with self-criticism: The role of compassionate self	Functional analysis of self-criticism Compassionate self-correction Using of the compassionate self in dealing with self-criticism How to manage fears, blocks and resistances to compassion	*Mindful breathing* *Functional analysis of self-criticism* *Compassionate self-correction* *Compassion for the Inner Critic*
**6**	Compassion always and everywhere	The multiple faces of compassion Compassionate behaviours Working with difficulties experienced with compassionate behaviour How to manage fears, blocks and resistances to compassion Compassionate planning Anticipating difficulties and setbacks in compassion engagement Manifesting compassion wherever we are and whenever we encounter suffering and difficulties (in self/others)	*Gratitude practice* *Mindfully remembering* *Self-compassion break* *The Compassion PDA* *Compassionate letter writing* *Compassionate flashcards* *Aspiration Wheel exercise*

Teachers attending the 8-week CMT-T were invited to practice the CMT-T exercises daily. In addition to self-report measures, teachers were asked to complete weekly Practice Diaries and Session Evaluation forms. Access to the Project website (https://escolascompassivas.wixsite.com/cmtescolas) allowed participants further information about the program, psychoeducation information and materials, practices instructions and audio files containing the meditation practices. Weekly gentle reminder emails were sent to participants motivating them to practice the exercises. A final CMT and Forest Bathing 1-day retreat was held at the end of the intervention.

The CMT-T team comprised five certified clinical psychologists with a PhD degree and CFT/CMT based clinical intervention experience and one teacher with a PhD degree, CFT training and certified mindfulness teacher training. Each training group had two lead facilitators, who facilitated the group, presented the content, led experiential exercises and promoted the discussion. All facilitators strictly followed the CMT-T manual and one of them guided all six groups. According to the BSP (2018) recommendations, the CMT-T team informed the participants that they could get in contact with the practitioners between the sessions in case they had any doubts or questions about the sessions’ contents.

### Waitlist control condition

Participants in the waiting list control group were not offered an intervention. After their parallel CMT-T group completed the intervention, participants in this group received the CMT-T intervention, and completed a post-intervention assessment (T3).

### Measures

*Demographics form*: Sociodemographic data were collected regarding age, marital status, teaching-related variables (e.g., education degree and teaching years).

#### Feasibility

Feasibility was assessed according to three criteria of Bowen et al. [96] framework: acceptability (defined as importance, relevance, and perceived helpfulness of sessions and exercises, as well as motivation and willingness to recommend the CMT-T to peers), practicality (assessed by program attendance and dropout), and adaptation (operationalized as adjustments made to the CMT-T curriculum from its pilot version [82].

#### Primary outcomes

*Types of Positive Affect Scale [TPAS; 97, 98].* The TPAS is a 18-items scale that measures the degree to which people experience different positive emotions. The scale assesses three different types of positive affect: activated positive affect (e.g., *excited*, *energetic*, *eager*), relaxed positive affect (e.g., *relaxed*, *calm*, *serene*), and safe positive affect (e.g., *safe*, *content*, *secure*). Each item/word is rated on a 5-point scale, and participants are asked to rate how characteristic each feeling is of them, ranging from *not characteristic of me* (0) to *very characteristic of me* (4). The Cronbach’s alphas for each subscale were .83 for relaxed and active positive affect and .73 for safeness/contentment positive affect [97]. In our sample, a Cronbach alpha of .74 was found for activated positive affect, .84 for relaxed positive affect and .81 for safe/content positive affect.

*Satisfaction with Teachers’ Professional Life [SWTPL; 99, 100].* The SWTPL is a 5-item scale aimed to assess global satisfaction with teachers’ professional life. Teachers rate each item by using a 5 Likert-type scale, ranging from *I completely disagree* (1) to *I completely agree* (5). In the original study, the SWTPL showed a Cronbach alpha of .91 [100]. In the current study, a Cronbach alpha of .83 was found.

*Depression, Anxiety and Stress Scales-21 [DASS-21; 101, 102].* The DASS-21 is a self-report instrument comprising three subscales that address depressive (seven items), anxiety (seven items) and stress symptoms (seven items). Participants are asked to rate the frequency of symptoms during the previous week using a 4‐point scale from *did not apply to me at all* (0) to *applied to me very much*, *or most of the time* (3). The Cronbach’s alphas for each subscale were .94 for depression, .87 for anxiety and .91 for stress [103]. In this study, Cronbach alpha values were .88 for depression, .84 for anxiety and .87 for stress.

*Shirom-Melamed Burnout Measure [SMBM; 104, 105].* The SMBM is a 14-items self-report measure addressing work-related burnout using a 7‐point scale, ranging from *never* (1) to *always* (7). The SMBM comprises three dimensions associated with work: physical exhaustion, cognitive weariness, and emotional exhaustion, with higher scores reflecting greater burnout symptoms. In the current study, the total score was used, and higher scores correspond to greater levels of burnout symptoms. Previous studies with the SMBM found excellent reliability [e.g., α = .96 for the total score; 106]. In the current study, a Cronbach alpha of .94 was found for the total score, and Cronbach alpha values were .93 for physical burnout, .96 for cognitive burnout and .86 for emotional burnout.

#### Secondary outcomes

*Compassionate Engagement and Action Scales [CEAS; 93, 107].* The CEAS assesses the three flows of compassion according to Gilbert’s evolutionary multidimensional model of compassion and the CFT framework: 1) Self-compassion, 2) Compassion for others, 3) Compassion from others. Each of these scales measures a) Compassionate Engagement (i.e., sensitivity to and motivation to engage with suffering) with items tapping into the competencies of sensitivity, sympathy, empathy, distress tolerance, non-judgment and care for wellbeing; and b) Compassionate Action (i.e., committed actions to try to alleviate and prevent suffering), with items focused on domains of helpful 1. attending, 2. thinking/reasoning, 3. behaving and 4. emotion/feeling. A definition of compassion is provided in the instructions of each scale. Items are rated according to the frequency of responding to one’s own suffering, others’ suffering or the experience of receiving compassion from others. A response scale ranging from *never* (1) to *always* (10) is used for rating the items. In the original study, all subscales presented acceptable to high reliabilities (range of .72 to .94) [93]. In the present study, the self-compassion subscale showed a Cronbach alpha of .90, the compassion for others subscale of .90, and the receiving compassion from others subscale of .96.

*Compassion Motivation and Action Scales [CMAS; 108, 109].* The CMAS encompasses two dimensions assessing self-compassion and compassion to others motivation and action and was developed based on a Motivational Interviewing approach to compassion. The two dimensions examine one’s desire, ability, reasons, and need for compassion to others and for oneself, as well as the commitment to compassionate and self-compassionate action. This 30-item self-report measure was designed to be specifically used as a measure of the change in compassionate motivation and action over time in clinical practice and intervention research. The self-compassion dimension encompasses 18 items and three subscales, (1) self-compassion intention, (2) self-compassion distress tolerance, and self-compassionate action. The compassion to others dimension includes 12 items tackling the same three subscales. A 7-point scale ranging from *completely disagree* (1) to *completely agree* (7) is used to rate each item. In the original study, the self-compassion scale presented a Cronbach alpha of .94 and the compassion to others Scale of .88. [108]. In the present study, the self-compassion scale showed a Cronbach alpha of .91 and the compassion to others scale of .85.

*Fears of Compassion Scale [FoC; 110, 111].* The FoC is a broadly used self-report measure of fears, blocks and resistances to compassion. It assesses barriers to giving compassion to others (10 items), receiving compassion from others (13 items), and being self-compassionate (15 items). The 38 items are answered on a 5-point Likert scale ranging from *don’t agree at all* (0) to *completely agree* (4). In the original study, Cronbach alphas were .85 for fear of compassion for self, .87 for fear of compassion from others and .78 for fear of compassion for others [110]. Cronbach alpha values for the fears of giving compassion to others, receiving compassion from others, and being self-compassionate were .87, .88, and .89, respectively.

*Forms of Self-Criticism and Self-Reassurance Scale [FSCRS; 112, 113].* The FSCRS is a 22-items self-report instrument assessing how one thinks and reacts when dealing with failures or setbacks. The FSCRS encompasses two forms of self-criticism: (1) inadequate-self and (2) hated-self and (3) assesses the ability to be self-reassuring. Each item follows the statement "When things go wrong for me. . .", and respondents are asked to choose in a 5-point scale, ranging from *not at all like me* (0) *to extremely like me* (4), the degree to which each item relates to their own experience. Additionally, in the current study, a self-criticism index was computed to the three subscales by summing the inadequate-self and hated-self subscales [SC-FSCRS; 114]. The original study revealed a Cronbach alpha of .87 for self-criticism. In the current sample, Cronbach alpha values were as follows: .80 for the inadequate-self, .67 for hated-self, .86 for the ability to self-reassure and .85 for the self-criticism index.

*Emotional Climate in Organizations Scales [ECOS; 115].* ECOS was developed based on the affect regulation systems model proposed by Gilbert [116] and assesses the presence/activation of the three affect regulation systems: threat, drive, soothing/safeness. Within three scales: 1) emotional climate, 2) satisfaction of needs, 3) motives underlying one’s actions. In the current study, only the emotional climate scale was used. Each scale comprises 15 items (five items for each type of emotion system), rated in a 5-point Likert-type scale scored between *never* (0) and *always* (4). Preliminary psychometric properties results suggest that the ECOS scales are valid and reliable: 1) Emotional Climate Scale: Threat α = .75, Drive α = .86, Safeness/soothing α = .83; Albuquerque et al., submitted manuscript). In the current study, the Emotional Climate Scale revealed Cronbach’ alpha values of .74, .84 and .81 for the threat, drive, and soothing/safeness scales.

#### Hear-rate variability

*Heart-rate variability assessment*. To control for possible confounding variables in the HRV analysis, participants completed a set of health-related questions before each HRV measurement, including height and weight (for BMI calculation), history/presence of cardiovascular or circulatory diseases, current medication intake, smoking habits (’are you a smoker?’ Yes/No), food intake (in the previous 2h), caffeine and alcohol intake (in the previous 4h), vigorous physical exercise (in the past 24h). For the measurement of HRV, the electrocardiogram (ECG) was recorded using Firstbeat Bodyguard2 (Firstbeat Technologies Ltd.) with a standard electrode configuration (right clavicle and precordial site V6). Two disposable Ag-AgCl electrodes were used. Participants were hooked up with the electrocardiogram (ECG) and asked to relax in a seated position for 5 minutes in order to obtain a measure of resting-state heart rate variability (HRV). Raw data (R-R intervals) were then imported into Kubios (version 2.1, 2012, Biosignal Analysis and Medical Imaging Group, University of Kuopio, Finland, MATLAB). Successive R waves (identified by an automatic beat detection algorithm) were visually inspected, and any irregularities were edited using Kubios’ automatic artefact correction algorithm (the maximum level of artefact correction applied was 2%). Heart rate and a time domain measure of HRV (Root Mean Square Successive Difference; RMSSD) were then obtained for pre- and post-intervention in both groups using Kubios [117]. According to the Task Force guidelines, the RMSSD reflects the integrity of vagus nerve-mediated autonomic control of the heart [118].

### Sample size

Power analysis was calculated at *priori* using G*Power 3.1 for Analysis of Variance (ANOVA). Results indicated that a sample size of 27 per condition (*n* = 54) was needed, using a significance level of .05 and a power of 95% to detect significant fixed effects, main effects, and interaction effects, with a large effect size (*f* = 0.25). Anticipating a 20% dropout rate, 65 participants were needed. Different strategies were used to minimize drop out from the study, such as sending weekly e-mail reminders for daily practice and emails for completing the self-report questionnaires.

### Randomization and blinding

Blocked randomization in a 1:1 ratio (block size = 6) took place within each enrolled school following the baseline assessment. Using a computer-based random allocation sequence (www.random.org), eligible participants were randomly assigned to an experimental or to a control condition within each school by a member of the research team. Due to the stepped wedge design of the study, blinding of participants was not possible as they had to be informed whether they would start with the intervention immediately or after 2 months.

### Statistical analyses

All data analyses were performed using IBM SPSS Statistics for Windows (Version 27.0), and the alpha level was set at .05. Descriptive statistics were calculated to characterise participants’ demographic and professional data. Baseline differences between the Compassionate Mind Training for Teachers (CMT-T) intervention group and the waitlist control (WLC) group were examined for demographics and for outcome variables in the study. For continuous variables, independent samples *t-tests* were conducted, and for categorical variables, chi‐square tests were performed. Independent samples *t-tests* and repeated measures ANOVA assumptions were verified through skewness and kurtosis. No severe violation of normal distribution was found (|Sk| < 3 and |Ku| < 8–10) [119].

To examine mean differences between pre-intervention (T1) and post-intervention (T2) in the primary outcomes (teachers’ burnout, depression, anxiety, stress, positive affect, satisfaction with professional life), secondary outcomes (flows of compassion, self-compassion and compassion motivations and actions, self-criticism, fears of compassion) and HRV, a series of 2 (condition) × 2 (time) repeated measures analysis of variance (ANOVA) were performed (considering the CMT-T and the WLC group as the between-subjects factor) to test the hypothesis that differences between pre-intervention and post-intervention differ between conditions. A significant timexgroup interaction effect suggests that the differences found between questionnaire scores at pre-intervention and post-intervention vary according to the condition to which the participants belong to. Furthermore, in order to examine the mean differences in the outcome variables within each group, a series of repeated measures analyses of variance (ANOVAs) were conducted for each group comparing pre- and post-intervention. Sphericity assumption for the repeated measures ANOVAs was analysed through Mauchly’s W. Whenever this assumption was not verified, we used the Greenhouse-Geisser epsilon (ε < .75), which corresponds to a probability correction factor of the F-statistics’ significance [120]. For ANOVAs, effect sizes were calculated using partial eta square (ƞ^2^p) and were interpreted as follows: partial ƞ2 values of .01 small, .06 medium and .14 large effect sizes [121]. For independent and paired-samples *t-tests*, effect sizes were calculated using Cohen’s d, with values between .2 and .4 representing small effects; between .5 and .7 medium effects and above .8 large effects. A confidence interval of 95% was used in all the analyses.

In addition, to explore the role of self-criticism on the effects of the CMT-T intervention, baseline self-criticism was included as a covariate. Subsequently, in the CMT-T group, high and low self-criticism groups were generated based on 75/25 percentiles of self-criticism baseline scores (High self-criticism: Self-criticism_Total > 18; Low self-criticism: Self-criticism_Total < 10). Paired-samples *t*-tests were then computed to examine mean differences in primary and secondary outcomes between high and low self-critics in the CMT-T group between pre (T1) to post-intervention (T2). To examine potential differences in the magnitude of change from pre-to-post intervention between high and low self-critics, change scores (T2 –T1) were computed, and independent samples *t*-test were calculated.

A stepped wedge analysis was performed in the subgroup of participants who participated in the WLC group between T1 and T2 and then completed the CMT-T between T2 and T3. Repeated Measures ANOVAs were performed to test differences from baseline to pre-intervention and post-intervention in all outcome variables. Post-Hoc analyses were used to explore pairwise differences (baseline-to-pre-intervention; baseline-to-post-intervention, and pre-to-post-intervention).

In order to assess whether the post-CMT results were maintained, repeated measures ANOVAs were computed to test for mean differences between post-treatment (T2) and 3-months follow-up (T3). Finally, Pearson product-moment correlation analyses were calculated to explore whether the CMT-T would strengthen the associations between the three flows of compassion from pre- to post-intervention in all participants who completed the CMT-T (*N* = 103).

## Results

### CMT-T feasibility

#### Acceptability

The majority of teachers assessed the CMT-T as very/extremely important (93.8%, *n* = 91), found the sessions very/extremely relevant (95.9%, *n* = 93) and helpful (96.8%, *n* = 93), and the practices from moderately to extremely adequate (99%, *n* = 96). Most teachers were highly motivated to attend the training (86.6%, *n* = 84) and would recommend it to others (96.9%, *n* = 94). The sessions considered most helpful were Modules 3 (*Building a compassionate mind/self*; 20.9%), 4 (*Working with emotions*: *The role of compassionate self*; 19.35%), 5 (*Understanding and working with self-criticism*: *The role of compassionate self;* 16.6%) and 2 (*Understanding the functions of our emotions*: *The three circles model;* 14.85%), with 14.75% of teachers (29.5%) rating all modules as most helpful. The practices rated as most helpful were Soothing Rhythm Breathing (78.9%), Compassion for the self (73.7%), Building the compassionate self (63.2), Mindfulness (60%) and Safe Place Imagery (58.9%).

#### Practicality

Overall, the intervention had a high attendance rate. From the 103 participants that completed the CMT-T intervention (66 at time 1 plus 37 at time 2), 94 attended the majority (≥5) of the eight sessions (*M* = 6.45, *SD* = 1.46). The nine (11.25%) participants that dropped out from the intervention attended on average 1.3 sessions.

#### Adaptation

A pilot version of the CMT-T, comprising the same six modules delivered across six weekly sessions, was implemented in both Portugal and the UK, and its feasibility and impact were assessed with promising results (e.g., 87). Nevertheless, this pilot version evaluation suggested the intervention could be improved in a few aspects. Therefore, the CMT-T was revised in terms of the number of sessions (from 6 to 8) and the length of the sessions was increased (30 more min per session) to allow the content of the modules to be fully covered and participants to follow the sessions at a gentler pace. Moreover, the topic of self-criticism emerged as a relevant theme that influenced the impact of the CMT-T. Thus, in the revised version of the CMT-T, self-criticism was addressed in several sessions across the intervention (and not only in session 5).

### Baseline differences

Baseline differences between the groups were explored for all outcome measures. At baseline the WLC group revealed higher levels of burnout total (*t*(150) = -2.479, *p* = .027, Cohen’ *d* = 0.04), and depressive symptoms (*t*(150) = - 2.570, *p* = .011, Cohen’ *d* = 0.04), in comparison with the CMT-T group. All differences represent very small effect sizes. No differences at the onset of the study were found for any other study variable.

### Differences between groups in changes from pre-intervention to post-intervention

Repeated measures ANOVAs comparing the CMT-T and WLC groups between baseline (T1) and post-intervention (T2) (Table 3) showed significant direct main effects of time for self-compassion and compassion to others motivation and action, fears of compassion for others, burnout, depression, anxiety and stress, and safe, relaxed and activated positive affect. Medium-to-large effects sizes were found, except for burnout and stress, where effect sizes were small. A significant direct main effect of time for HRV (RMSSD) with a medium effect size was found. Significant direct group effects were found for compassion for self, compassion to others motivation and action, fears of compassion for self, self-criticism, intrapersonal mindfulness, safe and activated positive affect, depression and stress, with effect sizes ranging from small to medium.

**Table 3:** Means, standard deviations before (T1) and after (T2) the CMT-T, time main effect, group main effect and time group interaction effect.

		CMT-T Group (*N* = 66)		WLC Group (*N* = 46)		Time			Group			Time X Group		
Measures	Time	*M*	*SD*	*M*	*SD*	*F*	*p*	ƞ^2^p	*F*	*p*	ƞ^2^p	*F*	*p*	ƞ^2^p
**Compassion for Self (CEAS)**	T1	63.05	14.93	60.37	17.44	2.52	.115	.023	4.92	**.029**	.044	7.65	**.007**	**.066**
	T2	68.52	14.99	58.89	16.12									
**Compassion for Others (CEAS)**	T1	76.63	13.04	75.72	13.08	0.208	.649	.002	2.59	.110	.023	5.47	**.021**	**.048**
	T2	78.73	12.26	72.59	12.40									
**Compassion from Others (CEAS)**	T1	64.05	18.16	61.64	18.28	0.24	.625	.002	1.66	.201	.015	1.14	.287	.010
	T2	64.83	16.32	59.53	14.36									
**Self-Compassion Motivation & Action (CMAS)**	T1	89.71	14.31	87.21	10.93	51.39	**< .001**	**.318**	26.40	.000	.194	40.06	**< .001**	**.267**
	T2	107.18	12.57	88.30	12.31									
**Compassion to others Motivation & Action (CMAS)**	T1	58.42	10.41	56.63	9.08	47.28	**< .001**	**.303**	12.39	**.001**	.101	16.10	**< .001**	**.128**
	T2	69.24	9.80	59.50	10.40									
**Fears of Compassion for Self**	T1	7.47	7.51	9.13	7.69	1.66	.201	.015	7.42	.**008**	.063	5.83	**.017**	**.050**
	T2	4.76	7.11	9.96	8.22									
**Fears of Compassion for Others**	T1	13.89	7.04	12.35	6.52	18.86	**< .001**	**.146**	1.15	.287	.010	20.41	**< .001**	**.157**
	T2	8.39	7.23	12.46	6.67									
**Fears of Compassion From Others**	T1	10.14	7.59	11.18	8.28	0.94	.334	.009	2.95	.089	.26	4.51	**.036**	**.040**
	T2	8.28	7.88	11.93	8.84									
**Self-criticism**	T1	15.68	7.19	18.80	8.94	0.11	.743	.001	8.27	**.005**	.071	2.95	.089	.027
	T2	14.40	7.34	19.67	9.77									
**Safe PA**	T1	2.38	.70	2.29	.66	13.61	**< .001**	**.111**	6.16	.**015**	.053	11.10	**.001**	**.092**
	T2	2.81	.69	2.31	.71									
**Relaxed PA**	T1	2.18	.75	2.13	.83	12.80	**.001**	**.105**	3.17	.078	.028	9.17	**.003**	**.078**
	T2	2.62	.80	2.17	.81									
**Activated PA**	T1	2.64	.69	2.50	.74	9.95	**.002**	**.084**	4.77	.**031**	.042	6.62	**.011**	**.057**
	T2	2.94	.55	2.53	.75									
**Satisfaction with Professional Life**	T1	14.62	4.88	12.57	4.47	1.63	.205	.015	1.33	.251	.012	7.316	**.008**	**.063**
	T2	14.06	5.23	14.11	5.02									
**Burnout**	T1	48.26	16.32	54.47	15.17	4.72	**.032**	**.041**	3.38	.069	.030	0.95	.333	.009
	T2	46.89	14.11	50.89	16.56									
**Depression**	T1	2.94	3.13	4.72	4.49	13.44	**< .001**	**.109**	11.02	.**001**	.091	0.06	.803	.001
	T2	1.82	1.68	3.74	3,83									
**Anxiety**	T1	2.97	3.54	4.24	3.65	10.78	**.001**	**.089**	3.99	.048	.035	0.22	.643	.002
	T2	2.13	2.63	3.13	3.56									
**Stress**	T1	6.45	3.84	8.02	4.28	4.56	**.035**	**.040**	6.52	**.012**	.056	0.03	.867	.000
	T2	5.71	2.97	7.15	3.53									
**HRV (RMSSD in ms**^**2**^**)** CMT-T (*n* = 51); WLC (*n* = 36)	T1	31.48	21.94	32.49	18.98	5.29	**.024**	**.059**	0.18	.892	< .001	0.76	.386	.009
	T2	37.62	31.85	35.26	20.08									

*Note*: CMAS = Compassion Motivation and Action Scale; CEAS = Compassion Engagement and Action Scales; PA = Positive affect.

There was a significant medium-to-large effect of the intervention (i.e., time x group interaction effects) on compassion for self, self-compassion and compassion to others motivation and action, fears of compassion for others, safe, relaxed and activated positive affect, and satisfaction with professional life. Significant time x group interaction effects with small effect sizes were found for compassion for others and fears of compassion for self and from others.

### Differences within groups in changes from pre-intervention to post-intervention

Mean differences from pre- to post-intervention in the study variables were then examined within each group through repeated measured ANOVAs. According to Table 4, when comparing mean scores in the CMT-T group before (T1) and after the program completion (T2), regarding those variables with significant time x group effects, significant increases were found in compassion for self, self-compassion motivation and action, compassion to others motivation and action, and in safe, relaxed and activated positive affect. Furthermore, there were significant decreases in fears of compassion for self, for others and from others. Results regarding those variables where no significant time x group effects were found, revealed that in the CMT-T group, there was also a significant decrease in depression and anxiety symptoms. The same analysis was conducted in the WLC group, a significant decrease was found for compassion for others, along with an increase in satisfaction with professional life, from T1 to T2. Significant decreases were also found for burnout and anxiety symptoms.

**Table 4:** Mean comparisons at T1 and T2 in the CMT-T and the in WLC group.

	CMT-T Group _T1 (*N* = 66)		CMT-T Group_T2 (*N* = 66)					WLC Group_T1 (*N* = 46)		WLC Group_T2 (*N* = 46)				
	*M*	*SD*	*M*	*SD*	*F* (65)	*p*	Ƞ^2^p	*M*	*SD*	*M*	*SD*	*F* (45)	*p*	Ƞ^2^p
**Compassion for Self (CEAS)**	63.05	14.93	68.52	14.99	9.71	**.003**	**.134**	60.37	17.44	58.89	16.12	.78	.382	.017
**Compassion for Others (CEAS)**	76.63	13.04	78.73	12.26	1.80	.184	.028	75.72	13.08	72.59	12.40	4.46	**.040**	**.090**
**Compassion from Others (CEAS)**	64.05	18.16	64.83	16.32	0.20	.655	.003	61.64	18.28	59.53	14.36	1.06	.309	.024
**Self-Compassion Motivation & Action (CMAS)**	89.71	14.31	107.18	12.57	83.96	**< .001**	**.564**	87.21	10.93	88.30	12.31	0.56	.459	.012
**Compassion to others Motivation & Action (CMAS)**	58.42	10.41	69.24	9.80	74.47	**< .001**	**.534**	56.63	9.08	59.50	10.40	3.43	.070	.071
**Fears of Compassion for Self**	7.47	7.51	4.76	7.11	8.18	**.006**	**.112**	9.13	7.69	9.96	8.22	0.56	.460	.012
**Fears of Compassion for Others**	13.89	7.04	8.39	7.23	44.96	**< .001**	**.409**	12.35	6.52	12.46	6.67	0.01	.905	.000
**Fears of Compassion From Others**	10.14	7.59	8.28	7.88	5.51	**.022**	**.078**	11.18	8.28	11.93	8.84	0.62	.434	.014
**Self-criticism**	15.68	7.19	14.40	7.34	2.22	.141	.034	18.80	8.94	19.67	9.77	1.04	.313	.023
**Safe PA**	2.38	.70	2.81	.69	28.65	**< .001**	**.309**	2.29	.66	2.31	.71	0.06	.811	.001
**Relaxed PA**	2.18	.75	2.62	.80	25.47	**< .001**	**.285**	2.13	.83	2.17	.81	0.14	.715	.003
**Activated PA**	2.64	.69	2.94	.55	24.87	**< .001**	**.280**	2.50	.74	2.53	.75	0.11	.739	.002
**Satisfaction with Professional Life**	14.62	4.88	14.06	5.23	1.34	.252	.020	12.57	4.47	14.11	5.02	6.08	**.018**	**.119**
**Burnout**	48.26	16.32	46.89	14.11	0.83	.367	.013	54.47	15.17	50.89	16.56	4.66	**.036**	**.096**
**Depression**	2.94	3.13	1.82	1.68	11.21	**.001**	**.147**	4.72	4.49	3.74	3.83	3.99	.053	.081
**Anxiety**	2.97	3.54	2.13	2.63	4.99	**.029**	**.071**	4.24	3.65	3.13	3.56	5.70	**.021**	**.112**
**Stress**	6.45	3.84	5.71	2.97	2.19	.144	.033	8.02	4.28	7.15	3.53	2.53	.118	.053
**HRV (RMSSD in ms**^**2**^**)** CMT-T (*n* = 51); WLC (*n* = 36)	31.48	21.94	37.62	31.85	**5.96**	**.018**	**.107**	32.49	18.98	35.26	20.08	.894	.351	.025

*Note*: CMAS = Compassion Motivation and Action Scale; CEAS = Compassion Engagement and Action Scales; PA = Positive affect.

**Fig 2:**
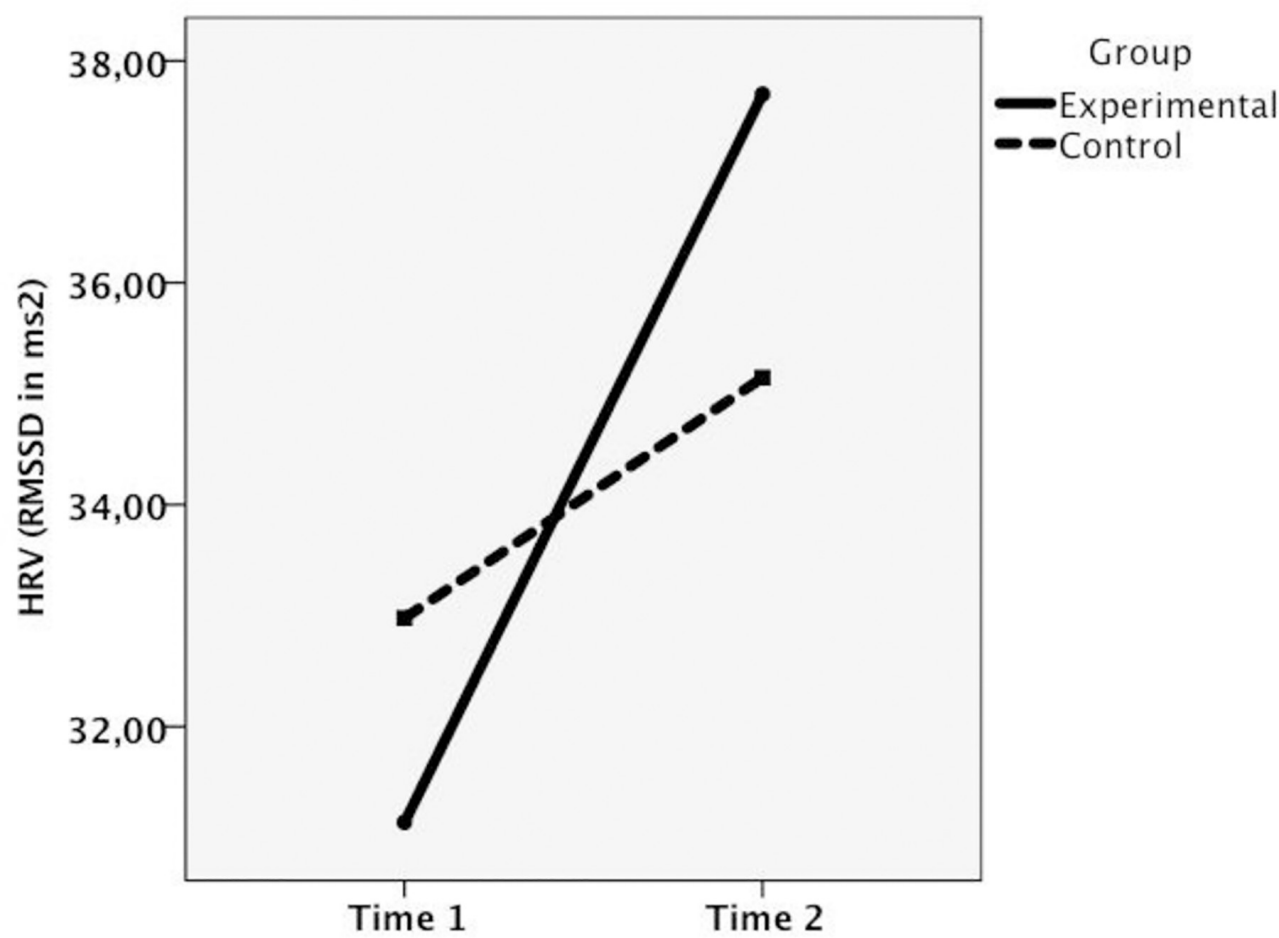
Time × group interaction for HRV (RMSSD; ms2).

We did not find a significant time x group interaction effect on HRV. However, given the reduced number of participants undertaking HRV measurements which might have impacted on the significance of the time x group interaction, the medium effect size found for the direct main effects of time, and a clear trend emerging by the visual inspection of the plot (Fig 2), we exploratively inspected mean differences at HRV (RMSSD) before (T1) and after the CMT-T (T2) in the two groups, separately. Repeated measured ANOVA showed a significant increase in HRV from T1 to T2, with a large effect size, only in the experimental group. Interestingly, the WLC showed a non-significant increase in HRV from T1 to T2 (see Table 4).

### The role of self-criticism

When controlling for baseline self-criticism, a significant time x self-criticism interaction effect was found (*F* = 1.90, *p* = .020, η^2^p = .368). In addition, significant effects of the intervention (i.e., time x group effects) were found for: compassion for self (*F* = 10.24, *p* = .002, η^2^p = .095), compassion for others (*F* = 4.33, *p* = .040, η^2^p = .043), self-compassion motivation and action (*F* = 47.22, *p* < .001, η^2^p = .327), compassion to others motivation and action (*F* = 14.37, *p* < .001, η^2^p = .129), fears of compassion for self (*F* = 5.41, *p* = .022, η^2^p = .053) and for others (*F* = 15.36, *p* < .001, η^2^p = .137), as well as safe (*F* = 9.23, *p* = .003, η^2^p = .087), relaxed (*F* = 8.48, *p* = .004, η^2^p = .080) and activated (*F* = 5.73, *p* = .019, η^2^p = .056) positive affect, and satisfaction with professional life (*F* = 5.35, *p* = .023, η^2^p = .052).

Paired-samples T-tests comparing high and low self-critics in the CMT-T group between baseline (T1) and post-intervention (T2) were then performed (Table 5). The high self-critics showed a significant increase in compassion for self, self-compassion motivation and action, compassion to others motivation and action, and in safe, relaxed and activated positive affect. These participants also revealed a significant decrease in fears of compassion for self and for others, in self-criticism and in depression and anxiety symptoms. As for the low self-critics, they showed a significant increase in self-compassion and compassion to others motivations and actions, and in safe, relaxed and activated positive affect, as well as significant decreases in fears of compassion for others. All effect sizes ranged from medium to large.

**Table 5:** Mean comparisons at T1 and T2 for the high self-criticism group (*N* = 31) and the low self-criticism group (*N* = 18) within the CMT-T group.

	High Self-criticism _T1		High Self-criticism _T2					Low Self-criticism _T1		Low Self-criticism _T2				
	*M*	*SD*	*M*	*SD*	*t*	*p*	*d*	*M*	*SD*	*M*	*SD*	*t*	*p*	*d*
**Compassion for Self (CEAS)**	60.80	16.75	67.27	14.80	-2.17	**.038**	**.40**	67.59	12.88	69.94	15.25	-0.70	.495	.17
**Compassion for Others (CEAS)**	76.24	14.24	79.04	13.54	-1.17	.253	.22	79.67	13.23	77.00	13.80	0.82	.426	.19
**Compassion from Others (CEAS)**	61.60	18.08	60.80	16.96	0.32	.750	.06	72.50	15.62	68.56	15.19	1.32	.204	.25
**Self-Compassion Motivation & Action (CMAS)**	87.32	15.43	108.58	12.03	-7.07	**< .001**	**1.27**	91.39	11.29	104.39	14.39	-3.52	**.003**	**.83**
**Compassion to Others Motivation & Action (CMAS)**	59.16	11.37	70.45	9.15	.6.11	**< .001**	**1.10**	55.72	9.70	66.22	11.99	-3.79	**.001**	**.93**
**Fears of Compassion for Self**	10.45	8.69	6.16	9.36	2.27	**.031**	**.41**	3.89	4.52	2.33	2.89	2.04	.058	.48
**Fears of Compassion for Others**	15.39	6.13	10.06	7.81	4.95	**< .001**	**.89**	11.28	7.78	7.50	6.37	2.92	**.010**	**.69**
**Fears of Compassion from Others**	12.90	8.22	10.29	9.72	1.66	.108	.30	6.17	5.93	5.22	4.67	0.91	.375	.22
**Self-Criticism**	21.83	4.80	17.80	6.89	3.17	**.004**	**.58**	7.28	2.52	9.89	5.95	-2.34	**.032**	**.55**
**Safe PA**	2.14	.68	2.63	.65	-3.90	**.001**	**.72**	2.72	.78	3.06	.70	-3.06	**.007**	**.74**
**Relaxed PA**	2.05	.91	2.66	.77	-4.86	**< .001**	**.88**	2.34	.62	2.68	.86	-2.39	**.029**	**.56**
**Activated PA**	2.45	.77	2.86	.56	-4.74	**< .001**	**.86**	2.82	.65	3.08	.55	-2.39	**.029**	**.56**
**Satisfaction with Professional Life**	13.77	5.12	13.77	5.59	0.00	1.00	.00	15.88	5.28	15.53	5.77	.536	.599	.13
**Burnout**	54.65	14.80	53.23	13.33	.631	.533	.11	43.67	17.43	40.78	13.99	0.97	.348	.23
**Depression**	4.03	3.54	2.23	1.54	3.15	**.004**	**.56**	2.00	2.77	1.17	1.29	1.59	.131	.39
**Anxiety**	4.35	4.23	2.74	3.31	2.62	**.014**	**.47**	1.50	1.69	1.78	1.73	-0.55	.593	.13
**Stress**	7.29	4.25	6.48	3.20	1.02	.320	.18	6.28	3.34	5.06	2.64	1.41	.178	.33

*Note*: CMAS = Compassion Motivation and Action Scale; CEAS = Compassion Engagement and Action Scales; PA = Positive affect.

To examine potential differences in the magnitude of change from pre-to-post intervention between high and low self-critics, change scores (T2 –T1) were computed, and independent samples *t*-test’s calculated. Results revealed no significant differences between high and low self-critics in the magnitude of change in all study variables, with the exception of anxiety [*t*_*(47)*_ = -2.10, *p* = .041] and self-criticism [*t*_*(46)*_ = -3.57, *p* = .001], where the change was greater in the high self-criticism group.

### Stepped wedge analysis: Differences in changes from baseline, pre-intervention to post-intervention within the WLC group’ participants who completed the CMT-T

Repeated Measures ANOVAs were performed to test differences in all study’s variables from baseline to pre-intervention and post-intervention (Table 6) in the WLC group participants who completed the CMT-T between T2 and T3. At post-intervention, participants presented increased levels of the three flows of compassion (for self, for others and from others), self-compassion and compassion to others motivations and actions, positive affect (safe, relaxed, activated) and satisfaction with professional life, as well as decreased fears of compassion (for self, for others and from others), burnout, depression, anxiety and stress symptoms. All differences reflected large effect sizes.

**Table 6:** Means and SDs of the outcome measures at baseline (T1), pre-intervention (T2) and post-intervention (T3) and repeated measures analysis of variance (*N* = 37) and pairwise comparisons, for the WLC group participants who completed the CMT-T intervention between T2 and T3.

	*Baseline (T1)*	*Pre-intervention (T2)*	*Post-Intervention (T3)*	*F*	*p*	*ƞ* ^*2*^ *p*	*Pairwise Comparisons*					
Outcome measures	*M (SD)*	*M (SD)*	*M (SD)*				*T1-T2*	*p*	*T1-T3*	*p*	*T2-T3*	*p*
**Compassion for Self (CEAS)** ^a^	59.66 (16.51)	59.22 (15.79)	68.38 (16.12)	9.84	**.001**	**.241**	.438 (1.79)	.809	-8.72 (2.83)	**.004**	-9.16 (2.25)	**< .001**
**Compassion for Others (CEAS)**	74.66 (14.52)	73.06 (13.19)	77.75 (12.66)	3.68	**.031**	**.106**	1.59 (1.76)	.372	-3.09 (1.82)	.100	-4.69 (1.69)	**.009**
**Compassion from Others (CEAS)** ^a^	60.06 (18.55)	58.63 (15.02)	68.50 (16.61)	7.10	**.004**	**.186**	1.44 (2.43)	.559	-8.44 (3.46)	**.021**	-9.88 (2.49)	**< .001**
**Fears of Compassion for Self**	9.84 (8.72)	10.66 (8.48)	5.28 (6.99)	9.31	**< .001**	**.231**	-.813 (1.35)	.551	4.56 (1.40)	**.003**	5.38 (1.28)	**< .001**
**Fears of Compassion for Others**	13.28 (6.91)	12.66 (7.20)	6.31 (5.41)	24.45	**< .001**	**.441**	.625 (.997)	.527	6.97 (1.20)	**< .001**	6.34 (1.12)	**< .001**
**Fears of Compassion From Others**	12.66 (8.94)	12.47 (9.40)	8.50 (7.79)	6.84	**.002**	**.181**	.188 (1.19)	.876	4.16 (1.31)	**.003**	3.97 (1.30)	**.005**
**Self-Compassion Motivation & Action (CMAS)**	87.13 (11.39)	88.25 (11.64)	108.06 (13.39)	70.09	**< .001**	**.693**	-1.13 (1.79)	.535	-20.94 (2.01)	**< .001**	-19.81 (2.15)	**< .001**
**Compassion to others Motivation & Action (CMAS)**	55.84 (9.16)	59.25 (9.63)	71.16 (9.01)	46.02	**< .001**	**.598**	-3.31 (1.82)	.071	-15.31 (1.61)	**< .001**	-11.91 (1.58)	**< .001**
**Self-criticism** ^a^	17.75 (8.53)	18.72 (9.16)	18.08 (10.82)	.310	.664	.010	-.97 (.94)	.311	-.34 (1.58)	.829	.63 (1.14)	.587
**Burnout** ^a^	53.75 (14.99)	50.28 (17.39)	43.22 (15.56)	9.53	**.001**	**.235**	3.47 (1.75)	.056	10.53 (2.71)	**.001**	7.06 (2.78)	**.016**
**Depression** ^a^	4.97 (4.76)	3.28 (3.43)	2.38 (2.59)	8.59	**.001**	**.217**	1.69 (.57)	**.006**	2.59 (.78)	**.002**	.91 (.52)	.092
**Anxiety**	4.16 (3.73)	2.53 (3.03)	2.09 (2.25)	8.73	**.001**	**.220**	1.63 (.53)	**.004**	2.06 (.55)	**.001**	.44 (.48)	.370
**Stress**	8.28 (4.22)	7.13 (3.77)	6.50 (2.65)	3.32	**.043**	**.097**	1.16 (.64)	.081	1.78 (.79)	**.031**	.63 (.66)	.354
**Safe PA**	2.33 (.74)	2.40 (.71)	2.78 (.76)	9.55	**< .001**	**.235**	-.07 (.11)	.517	-.45 (.12)	**.001**	-.38 (.10)	**.001**
**Relaxed PA**	2.10 (.76)	2.18 (.80)	2.70 (.83)	13.08	**< .001**	**.297**	-.08 (.11)	.492	-.60 (.12)	**< .001**	-.52 (.15)	**.002**
**Activated PA**	2.53 (.84)	2.59 (.83)	2.79 (.79)	3.95	**.024**	**.113**	-.06 (.09)	.531	-.26 (.10)	**.017**	-.20 (.09)	**.045**
**Satisfaction with Professional Life**	13.41 (4.43)	14.94 (5.04)	15.78 (4.50)	6.13	**.004**	**.165**	-1.53 (.79)	.063	-2.36 (.60)	**< .001**	-.84 (.65)	.204
**Threat Emotions**	-	6.25 (2.89)	5.72 (2.80)	3.61	.065	.094	-	-	-	-	.53 (.28)	.065
**Soothing Emotions**	-	13.31 (2.75)	14.06 (2.46)	3.97	**.050**	**.102**	-	-	-	-	-.75 (.38)	**.050**
**Drive Emotions**	-	11.92 (3.31)	13.33 (3.22)	13.69	**.001**	**.281**	-	-	-	-	-1.42 (.38)	**.001**
**HRV (RMSSD in ms** ^**2**^ **; n = 36)**	32.49 (18.98)	35.25 (20.08)	35.04 (17.85)	.46	.631	.010	-2.76 (17.55)	.351	.21 (21.07)	.952	-2.55 (18.73)	.419

Note: ^a^ = Greenhouse-Geisser correction; CMAS = Compassion Motivation and Action Scale; CEAS = Compassion Engagement and Action Scales; PA = Positive affect.

Furthermore, post-hoc pairwise comparisons revealed no significant changes from baseline to pre-intervention in any of the study variables except for symptoms of depression and anxiety, which decreased between T1 and T2. Between baseline and post-intervention, participants showed significant improvements in self-compassion and compassion from others, self-compassion and compassion to others motivations and actions, positive affect (safe, relaxed, activated) and satisfaction with professional life, along with significant reductions in fears of compassion (for self, for others and from others), burnout, and depression, anxiety and stress symptoms. In addition, between pre-intervention and post-intervention, participants revealed significant increases in the three flows of compassion (for self, for others and from others), in self-compassion and compassion to others motivations and actions and in positive affect (safe, relaxed, activated). They also revealed significant decreases in fears of compassion (for self, for others and from others) and burnout. The emotional climate at work was also assessed in these participants at T2 and T3, and significant increases in soothing/safeness and drive/vitality emotions at work were found from pre-to-post-intervention. A decrease in threat emotions at work was also observed, but did not reach the significance threshold.

### Three-month follow-up comparison with post-intervention

Repeated measures ANOVAs results considering post-CMT-T (T2) and the 3-months follow-up (T3) are presented in Table 7. No significant differences were found for all the considered variables pointing to the maintenance of the CMT-T therapeutic gains, except for self-compassion motivations and actions and compassion (to others) motivations and actions. Pairwise comparisons showed a significant decrease from T2 to T3 in the self-compassion motivations and actions (*p* < .001) and in the compassion to others motivations and actions (*p* = .020).

**Table 7:** Mean comparisons at post-CMT-T (T3) and 3-months follow-up (T4), effect size and observed power (*N* = 48).

	T3		T4					
Outcome measures	*M*	*SD*	*M*	*SD*	*F* (1, 46)	*p*	ƞ^2^p	Observ. power
**Compassion for Self (CEAS)**	69.35	13.92	66.89	13.10	2.93	.094	.06	.389
**Compassion for Others (CEAS)**	78.33	12.26	77.16	12.51	0.84	.366	.02	.145
**Compassion from Others (CEAS)**	63.22	16.41	60.57	14.04	2.02	.162	.04	.285
**Self-Compassion Motivation & Action (CMAS)**	109.72	10.10	102.72	13.05	14.52	**< .001**	.24	.961
**Compassion to others Motivation & Action (CMAS)**	71.49	9.75	67.37	11.12	5.82	**.020**	.12	.654
**Fears of Compassion for Self**	6.28	8.70	8.19	12.28	2.03	.161	.04	.286
**Fears of Compassion for Others**	8.74	7.79	10.02	9.45	2.24	.141	.05	.311
**Fears of Compassion From Others**	9.32	8.73	10.11	10.98	0.40	.532	.01	.095
**Self-criticism**	16.88	9.50	15.84	9.90	0.64	.427	.02	.123
**Safe PA**	2.76	0.75	2.70	0.67	0.32	.572	.01	.086
**Relaxed PA**	2.78	0.63	2.76	0.69	0.02	.884	.00	.052
**Activated PA**	2.82	0.63	2.87	0.64	0.46	.499	.01	.102
**Satisfaction with Professional Life**	15.33	4.44	15.59	4.45	0.26	.614	.01	.079
**Burnout**	43.59	12.35	45.02	16.49	0.52	.476	.01	.108
**Depression**	2.30	2.26	2.93	3.83	1.33	.255	.03	.204
**Anxiety**	2.45	2.64	2.43	3.18	0.00	.957	.00	.050
**Stress**	6.29	2.69	5.47	3.63	3.18	.081	.07	.415
**Threat Emotions**	5.48	2.65	5.08	2.66	1.02	.322	.04	.163
**Soothing Emotions**	13.94	2.30	13.88	1.93	0.05	.835	.00	.055
**Drive Emotions**	13.25	2.69	13.21	2.32	0.01	.929	.00	.051

*Note*: CMAS = Compassion Motivation and Action Scale; CEAS = Compassion Engagement and Action Scales; PA = Positive affect.

### How are the flows of compassion related pre- and post-intervention?

We explored whether the association between the flows of compassion changed from pre-to-post intervention in all participants who completed the CMT-T. Prior to the CMT-T, correlations between the three flows were moderate: *r*_Self-compassion—Compassion for Others_ = .54, *p* < .001; *r*_Self-compassion—Compassion From Others_ = .40, *p* < .001; *r*_Compassion for Others—Compassion From Others_ = .39, *p* < .001. After the intervention, correlations between the flows increased in magnitude across all the flows: *r*_Self-compassion—Compassion for Others_ = .60, *p* < .001; *r*_Self-compassion—Compassion From Others_ = .54, *p* < .001; *r*_Compassion for Others—Compassion From Others_ = .52, *p* < .001.

## Discussion

Schools are facing an unparalleled mental health crisis. Teachers within all education sectors and across countries increasingly reveal elevated stress and burnout and intend to leave the profession [3, 4]. This scenario is particularly concerning in Portugal, where teachers’ stress and burnout are prevalent and associated with the competitive pressures and growing retention crisis in the teaching profession [5]. Importantly, teachers’ stress has adverse consequences to their mental and physical health and negatively influences pupils [e.g., 5, 8]. Furthermore, this raises serious economic, healthcare and societal challenges. Therefore, it is crucial to promote adaptive cognitive and emotional regulation that supports teachers in coping with the challenges of the school context and promotes their mental wellbeing.

Growing empirical support has highlighted the beneficial impact of compassionate-based interventions on improving emotional regulation skills central to stress regulation [63]. In educational settings, the Compassionate Schools Research Initiative developed and examined the impact of a Compassion Mind Training intervention for Teachers (CMT-T), in schools in Portugal and the UK, and found empirical support for its international utility, feasibility and preliminary effectiveness on a range of mental health indicators [32, 87, 91]. The current study intended to expand this preliminary evidence and test the feasibility and effectiveness of a refined version of the CMT-T on teachers’ psychological distress, wellbeing, compassion to self and others, and heart rate variability (HRV), using a randomised controlled and stepped wedge design in a larger sample.

The CMT-T had a high attendance rate, and teachers considered the intervention very important and helpful. They were highly motivated to attend the sessions and would recommend it to colleagues. These findings suggest that the revised 8-week CMT-T was highly rated in terms of acceptability and revealed adequate practicality and adaptation, providing evidence that the CMT-T is a feasible intervention for teachers. These feasibility results are in support of previous pilot studies using an earlier version of the CMT-T in Portugal and the UK [32, 87, 91], reinforcing the acceptability of CMT-T modules and practices. In particular, teachers found the modules’ Building a compassionate mind/self’, ’Understanding and working with self-criticism’, and ’Understanding the functions of our emotions’ to be the most helpful. In general, this is similar to the results reported in the Portuguese pilot study [87]. In both studies, the two modules addressing the soothing system’s cultivation and the development of the compassionate mind/self and, multiple selves, were chosen by teachers as the most useful. Interestingly in this refined CMT-T, where self-criticism was targeted throughout the intervention, the module focused on the functional analysis of self-criticism and using the compassionate self to deal with it was also identified as a very relevant one. In line with Matos et al. [87], the practices assessed as the most helpful by the teachers were the Soothing Rhythm Breathing, Compassion for the self, Building the compassionate self, followed by Mindfulness and Safe Place Imagery. These acceptability results extend current knowledge on the evaluation of CMT interventions with community samples [e.g., 66, 67, 74] and should inform the development, implementation and evaluation of CMT interventions in future research.

The present randomised controlled study revealed significant time x group interaction effects of the CMT-T on compassion for self, self-compassion and compassion to others motivation and action, fears of compassion for others, safe, relaxed and activated positive affect, and satisfaction with professional life, with medium to large effect sizes. Moreover, significant time x group interaction effects with small effect sizes were also found for compassion for others and fears of compassion for self and from others. These findings partially support our hypotheses and are discussed in detail below concerning the process and outcome variables.

The results highlighted several differences between groups. Regarding the effects of the CMT-T on self-compassion, compared with the WLC group, teachers in the CMT-T group revealed a significant increase in compassion for self and self-compassion motivation and action and a significant reduction in fears of compassion for self. These changes represented medium to large effect sizes. Stepped wedge analyses further corroborated these findings revealing that teachers who received the intervention after acting as controls also showed significant improvements in compassion for self and self-compassion motivation and action, and significant decreases in fears of compassion for self after completing the CMT-T. These results corroborate our hypotheses, indicating that after the CMT-T, teachers improved their sensitivity and engagement with their own suffering, along with an enhanced motivation to engage with life’s difficulties and suffering with a caring and accepting attitude towards oneself, instead of withdrawing, avoiding or denying those difficulties. They also revealed an increased ability to tolerate distress concerning oneself, to be kind and supportive when facing hardships, and showed a greater capacity to act compassionately towards themselves. Simultaneously, the CMT-T produced a decline in fears, blocks and resistances to be self-compassionate. These results extend those reported by Matos et al. [87], documenting significant increases in teachers’ self-compassion motivation and action (as assessed by the CMAS) after the CMT-T, but where changes in self-compassion attributes and competencies (as measured by the CEAS), and fears of compassion for self, did not reach statistical significance. In the present study, significant improvements in compassion engagement and action towards oneself (as measured by the CEAS) were additionally found in teachers at post-intervention, which is in line with previous studies showing similar results using CMT in community samples [66, 67]. This finding suggests that this refined longer version of CMT-T not only promotes an increase in one’s motivation to be accepting and caring, to tolerate distress, and to commit to behaving compassionately towards oneself (as assessed by the CMAS), but also diminishes the inhibitors to be self-compassionate, and fosters the sensitivity to and engagement with one’s suffering including competencies of sensitivity, sympathy, empathy, distress tolerance, non-judgment and care for wellbeing (i.e., self-compassionate engagement) and committed actions to try to alleviate and prevent one’s suffering (i.e., self-compassionate action). Our results go beyond those of an earlier version of the CMT-T where improvements in self-compassion [as measured by the Self-Compassion Scale, SCS; 122] were only significant with increased practice of the techniques introduced [and not just session attendance), as supported by the qualitative analyses [32]. Furthermore, these findings are also in support of studies using CMT in other professions that found significant increases in self-compassion (as measured by the SCS) in health care educators and providers [68], mental health professionals [69], psychotherapy students [73], and firefighters [72].

The CMT-T also targets the cultivation of compassion for others [58, 61], and participants in the CMT-T group revealed significant increases in compassion to others motivation and action (as measured by the CMAS) and a significant reduction in fears of compassion for others, from pre to post-intervention, with medium to large effect sizes. These findings partially support our hypotheses and indicate that the CMT-T seems to reduce teachers’ fears, blocks and resistances of being compassionate to others while also promoting their motivation, distress tolerance, and commitment to act in compassionate ways towards others. Even though there was a trend towards positive change in compassion for others engagement and action (as measured by the CEAS), this increase did not reach the threshold of significance, contrarily to what was found in the CMT-T pilot study [87]. This finding might be attributable to a ceiling effect, which has been reported in previous studies with this measure [93] and using CMT in community samples [66, 67] as participants’ baseline scores were higher in compassion for others, in comparison to the other two flows of compassion, which might be related to a social desirability bias. In fact, results from the stepped wedge analyses support this hypothesis and show that, after receiving the CMT-T intervention, WLC participants also exhibited significant increases in compassion for others (as measured by the CEAS) as well as in compassion to others motivation and action and significant decreases in fears of compassion for others. A more in-depth observation of these findings revealed that even though the post-intervention scores of the CMT-T and the WLC participants who received the intervention between T2 and T3 were similar, the former presented baseline levels higher than the pre-intervention scores of the latter. In fact, in group comparisons analyses, the WLC group showed a significant decrease in compassion for others between baseline and pre-intervention (with a medium effect size). This was an unexpected finding, which might be related to the elevated baseline scores in this measure and to the parallel increase in burnout levels in these WLC participants. As a whole, the present results add to prior CMT studies with teachers where this flow of compassion was not specifically evaluated using a recognised quantitative measure [32] and with other professions [e.g., healthcare educators and professionals, 68, 69; fire service personnel, 72].

In regard to changes in compassion from others, teachers in the CMT-T group significantly decreased their fears of receiving compassion from others (with a medium effect size), although no significant differences were found in compassion received from others as measured by the CEAS, which assesses how one perceives other people’s motivation and ability to engage with one’s suffering and to take action to alleviate one’s distress. Stepped wedge analyses further add to these results, revealing significant increases in the perception of compassion received from others (as measured by the CEAS) and reductions in fears of compassion from others. These findings extend the ones described in the CMT-T pilot study [87], where neither compassion from others nor fears of receiving compassion from others significantly changed from pre- to post-intervention. In the current study, and as expected, the CMT-T produced a reduction in teachers’ inhibitors and resistances to being the recipient of compassion from others which is related to an improvement in one’s ability to be open and willing to receive compassion from others. Mixed results regarding CMT-T induced increases in the perception of others being more compassionate towards the self in this study warrant further exploration. Overall, our results are aligned with previous studies using CMT in community samples [66, 67] and add to preceding research implementing CMT with teachers [32, 91] and other professionals [68–70, 72, 73] that did not assess this flow of compassion.

An important aspect of the CFT/CMT approach is the consideration of the inter-relationship between the three flows of compassion (CEAS: compassion for others, being open to compassion from others, and self-compassion). In line with our hypothesis, results showed that the association between the three flows of compassion was strengthened after the CMT-T implementation. These data highlight that CMT-T seems to enhance the general level of several components of the three flows of compassion and reinforce engenderment of a compassionate mind in which higher scores in one flow of compassion tend to be accompanied by higher scores in another flow. These results are analogous to previous research exploring CMT in the general population [e.g., 66, 67] and provide further support to the assumption that CMT stimulates the caring motivational system, which facilitates one’s openness and motivation [58, 66, 116].

Taken together these results and the specificity of the target population, this study provides evidence for the effectiveness of the CMT-T in reducing teachers’ fears, blocks and resistances to compassion (for self, for others and from others) while also facilitating their motivation, distress tolerance and commitment to be compassionate towards themselves and others, along with developing the attributes and practicing the competencies of self-compassion and compassion for others. Therefore, the CMT-T may contribute to attenuating the barriers to compassion and strengthening teachers’ compassionate mind and abilities, including greater self-compassion, openness to receiving compassion and support from others, and motivation and competencies to establish more compassionate relationships, particularly in the school environment (e.g., colleagues, staff, pupils).

One of the central aims of CMT-T is to promote both overall and professional wellbeing, which in the current study were assessed through types of positive affect, linked to feelings of relaxation and calmness, feelings of safeness and contentment, and energised positive emotions (e.g., excited, energised, enthusiastic), and through teachers’ satisfaction with their professional life. Group comparisons revealed that the CMT-T group significantly increased safe, relaxed and activated positive affect from pre- to post-intervention, with large effect sizes. Stepped wedge analyses further substantiated these findings and revealed that, at post-intervention, WLC participants who completed the CMT-T presented incremented levels of feelings of safeness and relaxation (with large effect sizes) and of vitality/activation (medium effect sizes). These results are in line with our hypotheses and with preceding research that reported increases in positive affect, particularly in feelings of safeness, contentment, and relaxation, after a CMT intervention in community samples [66, 67]. Conversely to these previous studies where no changes were found in activated positive affect, interestingly, the CMT-T produced enhanced energizing positive emotions (e.g., excitement, vitality and enthusiasm) hypothesized to be related to the drive system, in addition to heightened positive emotions of safeness, contentment, calmness and relaxation, hypothesized to be related to the soothing system [58, 116]. These findings support the CFT framework regarding the beneficial impact of fostering compassion and reducing its inhibitors on cultivating types of positive affect linked to the affiliative and care-giving mentalities [58, 116]. Moreover, it may be that the specificity of the setting where CMT-T was implemented facilitates the promotion of a different type of active positive emotions related to the drive resource-seeking system [58, 116]. This may be related to the fact that teachers tend to suffer from burnout and exhaustion [4], which may be reflected in a dampening of this energizing positive affect prior to the intervention. Hence, the fact that CMT-T is applied to their professional lives and within the school context may encourage an adaptive stimulation of the drive resource-seeking system and foster this type of positive energizing emotions which, balanced by the promotion of feelings of safeness and relaxation, may be crucial to professional performance and wellbeing. In fact, this hypothesis seems to be further supported by the results using a new measure assessing emotional climate at work (i.e., the activation of the safeness, drive and threat affect systems) in a subsample of teachers. These participants exhibited significant increases in positive emotions linked to soothing/safeness and drive/vitality at work from pre-to-post CMT-T, representing medium to large effect sizes. Regarding teachers’ satisfaction with professional life, no significant changes were found in the CMT-T group. However, stepped wedge analyses revealed significant increases between baseline (T1) and post-intervention (T3) in the WLC participants who received the CMT-T (medium effect size). Of note, in the group comparison analyses, the WLC group presented a significant increase in satisfaction with professional life from T1 to T2. However, at baseline, these participants had lower scores in satisfaction with professional life than the CMT-T group, and this increase put both groups at a similar level at T2. As a whole, these results concerning the valuable effects of CMT-T on overall and professional wellbeing extend previous studies using earlier versions of CMT-T that did not assess these indicators [32, 87] and findings from research using CFT as guided self-help in the general population that demonstrated improvements in wellbeing at post-intervention [79].

Alongside the cultivation of positive emotions and wellbeing, the CMT-T also aims to target and reduce suffering and psychological distress. Although no significant interaction effects were found for outcome measures of psychological distress, within-group comparisons revealed that teachers in the CMT-T group demonstrated significantly decreased anxiety and depression symptoms (with medium and large effect sizes, respectively). Furthermore, stepped wedge analyses showed that, at post-intervention, WLC participants who completed the CMT-T presented decreased burnout, depression, anxiety and stress, all corresponding to large effect sizes. In relation to the WLC participants, a significant decrease in burnout and anxiety symptoms was found between baseline and post-intervention (medium effect sizes), even though their post-intervention scores were still higher than the CMT-T group baseline scores. This may be attributable to a ceiling effect given that at baseline, the WLC participants scored significantly higher than the CMT-T group in burnout and depression (small effect sizes). These findings partially corroborate our hypotheses, hinting at the potential benefits of the CMT-T in helping teachers diminish burnout and psychological distress. These results add to the CMT-T pilot study, which had already documented a decrease in psychopathological indicators at post-intervention but failed to find significant changes in burnout [87]. They also expand upon Maratos et al. [32] feasibility study, where thematic analyses pointed to the positive impact of CMT on dealing with emotional difficulties, although quantitative analyses did not find significant changes. Our findings are also in line with previous studies in community samples [66, 67] and other professionals [i.e., firefighters, 72] which attested the positive impacts of CMT and CFT in psychopathological indicators.

The present study also aimed to explore the impact of the CMT-T on heart rate variability (HRV), an indicator of vagal regulatory activity and a physiological marker of a person’s ability to flexibly respond to environmental challenges and regulate emotional responses [40, 50, 75], which has been proposed as a primary measure to assess and train compassion [44, 56]. Results showed that HRV significantly increased in the CMT-T group from baseline (T1) to post-intervention (T2). This suggests that, as previously found [67], CMT-T may produce an increase in vagal tone which is associated not only with the ability to downregulate physiological arousal, but also with the experience of inter- and intrapersonal safeness, and the inhibitory function of the prefrontal cortex with resulting greater capacity for emotion regulation [123], metacognitive awareness, and empathy. Relevantly, higher vagal tone has also been associated with better physical health: better glucose regulation, better HPA axis function, reduced inflammation, reduced risk for cardiovascular disease, and all-cause mortality [40]. However, surprisingly, the WLC group also demonstrated an increase in HRV (even though non-significant) from T1 to T2, which might be one of the reasons why the time x group interaction effect test did not reach statistical significance. These findings differ from previous investigations where only the intervention group showed significant changes in HRV [67]. One of the possible explanations of this result could be the beneficial effects of the CMT-T intervention not only on the group undertaking the intervention but also on the whole school climate. In fact, it is possible that the WLC participants have been positively impacted by the expectation of receiving a compassion focused intervention, or by believing that they work for and belong to an institution that promotes such initiatives that show interest and care for their psychological needs. Indeed, perceived positive organizational climate has been shown to be related to heart rate variability [124, 125]. Given the study design where teachers were randomised within their school into the CMT-T and WLC groups, it is also possible that WLC participants already started benefitting from the increased compassion the intervention group was developing as a result of the intervention. This alludes to the intriguing possibility that interventions leading to increased compassion related variables and physiological regulation in a group might potentially have a positive impact on other groups in the same institution, virtually initiating an upward spiral [126].

A psychological process that might play a role in how participants responded to the CMT-T is self-criticism. Self-criticism is a transdiagnostic and major vulnerability factor to mental health difficulties and poor wellbeing [127], being linked to a variety of adverse interpersonal (e.g., submissive behaviour, aggression) and intrapersonal (e.g., shame, rumination, worry, poor emotion regulation skills) factors in nonclinical populations [128, 129]. Importantly, self-criticism is known to influence the impact of compassion and self-compassion in wellbeing [67, 88]. In the current study, and contrarily to our hypothesis, no significant interaction effects of the CMT-T were found on self-criticism. Although previous research using CMT in community samples [66, 67, 79] and other professional groups [e.g., psychotherapy students, 73, healthcare educators and professionals, 68, 69] found changes in self-criticism, similar results to ours were reported in the pilot studies of CMT for teachers in Portugal [CMT-T; 87] and the UK [32]. Despite in the refined version of the CMT-T, implemented in the present study, self-criticism having been addressed throughout the sessions and specifically on module 5, the decreasing trend in self-criticism levels that could be observed in the CMT-T group did not reach statistical significance. Future iterations of the CMT-T should seek to clarify these findings and explore whether this resistance to change in self-criticism is attributable to the specificity of the target population and whether it is replicable in other samples of teachers. A possible aspect that could be adjusted in the CMT-T is exchanging the order of the modules so that the functional analysis of self-criticism and strategies to use the compassionate self to work with it (module 5 in the CMT-T) come earlier in the intervention.

Considering previous research emphasising that individual differences in self-criticism influence the impact of compassion-based interventions in general [88–90], and of the CMT-T in particular [87], we examined the role of self-criticism on the effects of the CMT-T intervention. When controlling for baseline self-criticism, a significant interaction (time x self-criticism) effect was found, and significant effects of the intervention (time x group) effects emerged in compassion for self and for others, self-compassion and compassion to others motivation and action, fears of compassion for self and for others, relaxed and activated positive affect, and satisfaction with professional life. These findings highlight that self-criticism seems to impact how teachers respond to CMT-T, playing a role in how the intervention operates in developing teachers’ abilities to be compassionate towards themselves and others, thus promoting their wellbeing and reducing psychological distress. In addition, when comparing changes between baseline and post-intervention in high and low self-critics of the CMT-T group, results showed that the high self-critics significantly improved compassion for self, self-compassion motivation and action, compassion to others motivation and action, and positive emotions of safeness, relaxation and activation, and decreased fears of compassion for self and for others, self-criticism and depression and anxiety symptoms. On the other hand, the low self-critics revealed significant increases in self-compassion and compassion to others motivations and actions, and in positive emotions (safeness, relaxation and activation), in addition to significant decreases in fears of compassion for others. All differences corresponded to medium to large effect sizes. Hence, high self-critics seem to benefit the most from the CMT-T revealing significant or greater reductions in fears of compassion, self-criticism, depression and anxiety, and improvements in self-compassion and compassion to others, and in positive emotions linked to safeness, relaxation and vitality, after the intervention compared to low self-critics. Indeed, the magnitude of improvement in anxiety and self-criticism was greater in the high self-critic participants. The current study extends the findings of the CMT-T pilot study [87], where self-criticism was found to influence the intervention effects on self-compassion, satisfaction with professional life and burnout. Our results underline the importance of addressing and working with self-criticism throughout a CMT intervention to facilitate changes in compassion, wellbeing and psychological distress. Future research implementing and testing the CMT-T or other CMT interventions should take into account these findings, in particular, considering specifically working with self-criticism ahead in the intervention whilst also targeting it across the sessions.

Importantly, in line with our hypothesis, the improvements seen in the CMT-T group and WLC group participants who received the intervention were largely maintained at three months follow-up, with the exception of self-compassion and compassion to others motivations and actions, which diminished. This reduction at follow-up might somehow be expected, given that the CMAS assesses one’s desire, reasons, and need for compassion to others and for oneself [108]. This means that if participants improvements in the flows of compassion were maintained from post-intervention to follow-up, then their perception of the need and motivation to continue to enhance their self-compassion and compassion to others might have faded away. However, only a longer follow-up period would allow us to confirm this hypothesis. Alternatively, it may be that an ongoing independent practice of CMT is needed to maintain its benefits on levels of self-compassion and compassion to others motivation and action, as typically encouraged by CMT practitioners. These results affirming the stability of the changes after the CMT-T add to prior studies using earlier versions of the CMT-T [32, 87] and are aligned with Irons and Heriot-Maitland (2020), who found a maintenance of CMT induced changes at three months follow-up in the general public, and with Sommers-Spikerman et al. [79] who described a retainment or amplification of the improvements after a guided self-help CFT in a community sample at three- and nine-months follow-up.

Taken together, our findings offer empirical support for the feasibility and effectiveness of the CMT-T on promoting teachers’ self-compassion and compassion for others, and overall and professional wellbeing, strengthening their physiological self-regulation via increased HRV, in addition to reducing their resistances to compassion and psychological distress. Specifically, the CMT-T seems to promote a shift from threat-focused competitive motives to more compassion affiliative focused ones in relation to both oneself and to others, in the sense that it lightens teachers’ fears and resistances to compassion whilst also enabling the development of their compassion abilities, motives and actions towards themselves and others. This may improve how teachers regulate their emotions and cope with the multiple challenges they encounter in their personal and professional lives that cause psychological distress [namely those related to competitive pressures focused on the self or others, achievement focus, performance evaluation, heavy workloads, pupils’ behavioural problems, 5, 36, 130]. The impact of the cultivation of such socio-emotional competencies and compassion motives seems to be reflected in teachers’ increased levels of safeness, relaxation and vitality positive emotions, and satisfaction with professional life, alongside decreased psychological distress symptoms found at post-CMT-T. The increased sense of intra- and inter-personal safeness promoted by the CMT-T was also reflected in the increased resting HRV reported by the participants in the experimental group.

As a whole, results from the current study highlight the utility and importance of implementing compassion-focused interventions in educational settings. The CMT-T may not only help teachers develop socio-emotional competencies for mental health and wellbeing but also contribute to cultivating a compassionate, prosocial and resilient culture in school settings. Therefore, the CMT-T might respond to key international guidelines for sustainable development prioritising the promotion of health and wellbeing, the cultivation of peaceful, resilient and inclusive societies, and the assurance of inclusive and equitable quality education for all [2]. At the same time, the CMT-T is aligned with national recommendations as it might help to achieve the goal of enhancing social-emotional competencies and health literacy in educational settings to foster health and wellbeing [81, 82].

### Limitations and future research

Despite the encouraging findings, the present study encloses some limitations that should be taken into consideration and addressed in future studies. First, due to the current trial design, participants could not be blinded to condition allocation, which may increase the risk of selection biases. Second, owing to the use of a waitlist control group, it is not possible to rule out the influence of nonspecific factors [131]. Third, because some of the CMT-T and WLC group participants were from the same school, potential contamination effects might have occurred. Fourth, the uneven gender distribution and overrepresentation of female teachers in our sample limit the generalizability of the findings. Fifth, although the use of a large sample and a randomised controlled stepped wedge design is a major strength of our study, effect sizes tend to be overestimated in RCT’s with waitlist-controlled designs, which may thus constraint the conclusiveness of our findings [132, 133]. Sixth, the control group remained on the waiting-list and did not receive another psychological intervention, and this limits conclusions on the effectiveness of the CMT-T over other interventions tailored for the same target population (e.g., CARE for teachers, CASEL). Additional trials with active comparison groups are necessary to provide more robust evidence of the efficacy of the CMT-T in comparison to other psychological interventions for teachers. Seventh, while teachers were instructed to practice the CMT exercises daily in-between each session and at-home practice as discussed at the beginning of each session, qualitative data regarding home-based practices were not analysed in the current study. Additionally, only a subsample of participants underwent the HRV assessment, which may thus limit the conclusiveness of our findings.

Furthermore, in light of past research indicating that practice frequency [32], practice helpfulness and the embodiment of the compassionate self may be crucial in promoting changes in a CMT intervention [74], it seems fruitful to investigate the role of these practice indicators on the effectiveness of the CMT-T. Previous studies have pointed to the mediating role of compassion and fears of compassion as key processes of change mediating the impact of CMT in teachers [87] and in general community samples [134], and thus future research should explore the processes that mediate changes from pre to post-intervention using the refined version of CMT-T. Future research is also warranted to shed further light on whether implementing the CMT-T on teachers would directly impact the quality of their relationships with pupils/parents or indirectly impact on pupils’ wellbeing, prosocial qualities, and academic performance. It is worth noting that the current study is part of a larger ongoing project, the Compassion in Schools Research Initiative, which aims at employing a systemic whole school approach, where the entire school community is involved (i.e., school boards, teachers, non-teaching staff, pupils and parents), and that aims at implementing tailored CMT programs to all educational agents and the pupils. Future studies resulting from this project will allow us to test the effectiveness of this whole-school approach. In fact, prior studies using CMT in organisational settings [e.g., mental health care services, 71] have emphasised the importance of adopting compassion approaches at all levels of an organisation. Finally, the low cost to deliver and usefulness of the CMT-T seems promising, and future work should continue to assess its effectiveness and promote its dissemination in other settings/countries, to establish the scalability of this intervention.

## Conclusion

The findings suggest that the CMT-T is a feasible and effective intervention to promote teachers’ self-compassion and compassion for others, their overall and professional wellbeing, psychophysiological self-regulation, as well as to reduce their psychological distress. In sum, CMT-T may not only enhance teachers’ wellbeing and reduce distress, thereby reducing individual suffering and relieving a substantial socioeconomic burden on society, but also contribute to creating safe, compassionate, collaborative, encouraging, and resilient educational environments for the benefit of all.

## Supporting information

S1 File CONSORT checklist. (DOC)

## References

[1] World Health Organization. (‎2013)‎. Mental health action plan 2013–2020 [Internet]. Geneva: World Health Organization; [cited 2021 Sept 10]. Available from: https://apps.who.int/iris/handle/10665/89966

[2] UN General Assembly [Internet]. New York (NY): Transforming our world: the 2030 Agenda for Sustainable Development, 21 October 2015, A/RES/70/1, available at: https://www.refworld.org/docid/57b6e3e44.html [accessed 9 September 2021]

[3] Jennings PA, DeMauro AA, Mischenko PP. Where are we now? Where are we going? Preparing our students for an uncertain future. In: Jennings PA, DeMauro AA, Mischenko PP, editors. The Mindful School. Transforming School Culture through Mindfulness and Compassion. New York (NY): Guildford Press; 2019. p. 3–13.

[4] Gray C, Wilcox G, Nordstokke D. Teacher mental health, school climate, inclusive education and student learning: A review. Can Psychol. 2017;58(3):203–210. doi: 10.1037/cap0000117

[5] Varela RC, della Santa R, Silveira H, Coimbra de Matos A, Rolo D, Areosa J. Inquérito nacional sobre as condições de vida e trabalho na educação em Portugal (INCVTE). Jornal da FENPROF. 2018 [cited 2021 Sept 10]. Available from: https://www.fenprof.pt/?aba=39&cat=667

[6] Skaalvik EM, Skaalvik S. Job satisfaction, stress and coping strategies in the teaching profession-What do teachers say? Int Educ Stud. 2015;8(3):181–192.

[7] Jennings PA, Greenberg MT. The prosocial classroom: Teacher social and emotional competence in relation to student and classroom outcomes. Rev Educ Res. 2009;79(1):491–525. doi: 10.3102/0034654308325693

[8] Betoret FD. Self‐efficacy, school resources, job stressors and burnout among Spanish primary and secondary school teachers: a structural equation approach. Educ Psychol (Lond). 2009;29(1):45–68. doi: 10.1080/01443410802459234

[9] McCallum F, Price D. Graham A, Morrison A. Teacher well-being: A review of the literature. Hawthorn (Australia): The Association of Independent Schools of New South Wales Limited; 2017 Oct 11. Available

[10] Naghieh A, Montgomery P, Bonell CP, Thompson M, Aber JL. (). Organisational interventions for improving wellbeing and reducing work‐related stress in teachers. Cochrane Database Syst Rev. 2015;(4). doi: 10.1002/14651858.CD010306.pub2 25851427 PMC10993096

[11] Stansfeld SA, Rasul FR, Head J, Singleton N. Occupation and mental health in a national UK survey. Soc Psychiatry Psychiatr Epidemiol. 2011;46(2):101–110. doi: 10.1007/s00127-009-0173-7 20033130 PMC3034883

[12] Education Support. Teacher Well-being Index. London (UK): Education Support; 2020. Available from: https://www.educationsupportpartnership.org.uk/sites/default/files/resources/teacher_wellbeing_index_2018.pdf [Last accessed 05.03.2020].

[13] NASUWT. (2016). The Big Question 2016: An opinion survey of teachers and school leaders. Available from: https://www.nasuwt.org.uk/uploads/assets/uploaded/7649b810-30c7-4e93-986b363487926b1d.pdf

[14] Sapolsky MR. Why zebras don’t get ulcers, 3rd ed. New York (NY): Holt Paperback; 2004.

[15] Heinrichs M, Baumgartner T, Kirschbaum C, Ehlert U. Social support and oxytocin interact to suppress cortisol and subjective responses to psychological stress. Biol Psychiatry. 2003;54:1389–1398. doi: 10.1016/s0006-3223(03)00465-7 14675803

[16] Alyamani RAS, Murgatroyd C. Epigenetic programming by early-life stress. Prog Mol Biol Transl Sci. 2018;157:133–150. doi: 10.1016/bs.pmbts.2018.01.004 29933948

[17] Ein-Dor T, Verbeke WJ, Mokry M, Vrtička P. Epigenetic modification of the oxytocin and glucocorticoid receptor genes is linked to attachment avoidance in young adults. Attach Hum Dev. 2018;20(4):439–454. doi: 10.1080/14616734.2018.1446451 29513137

[18] Hoglund WLG, Klingle KE, Hosan NE. Classroom risks and resources: Teacher burnout, classroom quality and children’s adjustment in high needs elementary schools. J Sch Psychol. 2015;53(5):337–357. doi: 10.1016/j.jsp.2015.06.002 26407833

[19] Harding S, Morris R, Gunnell D, Ford T, Hollingworth W, Tilling K, et al. Is teachers’ mental health and wellbeing associated with students’ mental health and wellbeing? J Affect Disord. 2019;253:460–466. doi: 10.1016/j.jad.2018.08.080 30189355

[20] Kidger J, Araya R, Donovan J, Gunnell D. The effect of the school environment on the emotional health of adolescents: a systematic review. Pediatrics. 2012;129(5):925–949. doi: 10.1542/peds.2011-2248 22473374

[21] Plenty S, Östberg V, Almquist YB, Augustine L, Modin B. Psychosocial working conditions: An analysis of emotional symptoms and conduct problems amongst adolescent students. J Adolesc. 2014;37(4):407–417. doi: 10.1016/j.adolescence.2014.03.008 24793388

[22] Oberle E, Schonert-Reichl KA. Stress contagion in the classroom? The link between classroom teacher burnout and morning cortisol in elementary school students. Soc Sci Med. 2016;159:30–37. doi: 10.1016/j.socscimed.2016.04.031 27156042

[23] McLean L, Connor CM. Depressive symptoms in third-grade teachers: Relations to classroom quality and student achievement. Child Dev. 2015;86(3):945–954. doi: 10.1111/cdev.12344 25676719 PMC4428950

[24] OECD iLibrary [Internet]. Paris: OCDE; 2021. Positive, high-achieving students?: What schools and teachers can do; [cited 2021 September 9]. Available from: 10.1787/3b9551db-en

[25] Frenzel AC, Goetz T, Lüdtke O, Pekrun R, Sutton RE. Emotional transmission in the classroom: Exploring the relationship between teacher and student enjoyment. J Educ Psychol. 2009;101(3):705–716. doi: 10.1037/a0014695

[26] Kunter M, Tsai YM, Klusmann U, Brunner M, Krauss S, Baumert J. Students’ and mathematics teachers’ perceptions of teacher enthusiasm and instruction. Learn Instr. 2008;18(5):468–482. doi: 10.1016/j.learninstruc.2008.06.008

[27] Cefai C, Bartolo PA, Cavioni V, Downes P. (2018). Strengthening Social and Emotional Education as a core curricular area across the EU. A review of the international evidence. Publications Office of the European Union. doi: 10.2766/664439

[28] Viac C, Fraser P. Teachers’ well-being: A framework for data collection and analysis. OECD Education Working Papers [Internet]. 2020 [cited 2021 Sept 10]; 213. Available from 10.1787/c36fc9d3-en2020

[29] Becker JC, Hartwich L, Haslam SA. Neoliberalism can reduce well‐being by promoting a sense of social disconnection, competition, and loneliness. Br J Soc Psychol. 2021;60(3):947–965. doi: 10.1111/bjso.12438 33416201

[30] Galton M, MacBeth J. Teachers under pressure. London: Sage Publications Ltd; 2008.

[31] Curran T, Hill AP. Perfectionism is increasing over time: A meta-analysis of birth cohort differences from 1989 to 2016. Psychol Bull. 2019;145(4):410–429. doi: 10.1037/bul0000138 29283599

[32] Maratos FA, Montague J, Ashra H, Welford M, Wood, Barnes C, et al. Evaluation of a Compassionate Mind Training intervention with school teachers and support staff. Mindfulness. 2019;10:2245–2258. doi: 10.1007/s12671-019-01185-9

[33] Rodway C, Tham SG, Ibrahim S, Turnbull P, Windfuhr K, Shaw J, et al. Suicide in children and young people in England: a consecutive case series. Lancet Psychiatry. 2016;3(8):751–759. doi: 10.1016/S2215-0366(16)30094-3 27236279

[34] Wetherall K, Robb KA, O’Connor RC. Social rank theory of depression: A systematic review of self-perceptions of social rank and their relationship with depressive symptoms and suicide risk. J Affect Disord. 2019;246:30–39. doi: 10.1016/j.jad.2018.12.045 30594043

[35] Cunha M, Matos M, Faria D, Zagalo S. (2012). Shame memories and psychopathology in adolescence: The mediator effect of shame. Rev Int Psicol Ter Psicol. 2012;12(2):203–218.

[36] Gilbert P, Matos M, Wood W, Maratos F. The compassionate mind and the conflicts between competing and caring: Implications for educating young minds. In: Coles MI, Gent B, editors. Education for survival: The pedagogy of compassion. Sterling (VA): Trentham Books; 2020. p. 44–76.

[37] Basran J, Pires C, Matos M, McEwan K, Gilbert P. Styles of leadership, fears of compassion, and competing to avoid inferiority. Front Psychol. 2019;9:2460. doi: 10.3389/fpsyg.2018.02460 30723443 PMC6349715

[38] Coles MI. Towards the compassionate school. From golden rule to golden thread. Sterling (VA): Trentham Books; 2015.

[39] Carter S, Bartal IB, Porges E. The roots of compassion: an evolutionary and neurobiological perspective. In: Seppälä EM, Simon-Thomas E, Brown SL, Worline MC, Cameron CD, Doty JR, editors. The Oxford handbook of compassion science. Oxford: Oxford University Press; 2017. p. 178–188.

[40] Petrocchi N, Cheli S. The social brain and heart rate variability: implications for psychotherapy. Clin Psychol Psychother. 2019;9:208–223. doi: 10.1111/papt.12224 30891894

[41] Gilbert P. Explorations into the nature and function of compassion. Curr Opin Psychol. 2019;28:108–114. doi: 10.1016/j.copsyc.2018.12.002 30639833

[42] Gilbert P, Choden. Mindful compassion. London: Constable Robinson; 2013.

[43] Crocker J, Canevello A. Consequences of self-image and compassionate goals. In: Devine PG, Plant A, editors. Advances in experimental social psychology. Amsterdam: Elsevier; 2012. p. 229–277.

[44] Di Bello M, Carnevali L, Petrocchi N, Thayer JF, Gilbert P, Ottaviani C. (2020). The compassionate vagus: A meta-analysis on the connection between compassion and heart rate variability. Neurosci Biobehav Rev. 2020;116:21–30. doi: 10.1016/j.neubiorev.2020.06.016 32554001

[45] Keltner D, Kogan A, Piff PK, Saturn SR. The sociocultural appraisals, values, and emotions (SAVE) framework of prosociality: Core processes from gene to meme. Annu Rev Psychol. 2014;65:425–460. doi: 10.1146/annurev-psych-010213-115054 24405363

[46] MacBeth A, Gumley A. Exploring compassion: A meta-analysis of the association between self-compassion and psychopathology. Clin Psychol Rev. 2012;32(6): 545–552. doi: 10.1016/j.cpr.2012.06.003 22796446

[47] Seppälä EM, Simon-Thomas S, Brown SL, Worline MC, Cameron CD, Doty JR. The Oxford handbook of compassion science. Oxford: Oxford University Press; 2017.

[48] Fredrickson BL, Grewen KM, Coffey KA, Algoe SB, Firestine AM, Arevalo JM, et al. A functional genomic perspective on human well-being. Proceedings of the National Academy of Sciences of the United States of America. 2013;110:13684–13689. doi: 10.1073/pnas.1305419110 PMC3746929 23898182

[49] Wang Y, Fan L, Zhu Y, Yang J, Wang C, Gu L, et al. Neurogenetic mechanisms of self-compassionate mindfulness: The role of oxytocin-receptor genes. Mindfulness. 2019;10:1792–1802. doi: 10.1007/s12671-019-01141-7

[50] Di Bello M, Ottaviani C, Petrocch N. Compassion is not a benzo: Distinctive associations of heart rate variability with its empathic and action components. Front Neurosci. 2021; doi: 10.3389/fnins.2021.617443 33776635 PMC7994334

[51] Kirby JN. Compassion interventions: The programmes, the evidence, and implications for research and practice. Psychol Psychother: Theory Res Pract. 2017;90:432–455. doi: 10.1111/papt 27664071

[52] Kirschner H, Kuyken W, Wright K, Roberts H, Brejcha C, Karl A. Soothing your heart and feeling connected: A new experimental paradigm to study the benefits of self-compassion. Clin Psychol Sci, 2019;7(3):545–565. doi: 10.1177/2167702618812438 32655984 PMC7324152

[53] Kim JJ, Parker SL, Doty JR, Cunnington R, Gilbert P, Kirby JN. Neurophysiological and behavioural markers of compassion. Sci Rep. 2020;10(1):1–9. doi: 10.1038/s41598-019-56847-4 32322008 PMC7176659

[54] Singer T, Engert V. It matters what you practice: Differential training effects on subjective experience, behavior, brain and body in the ReSource Project. Curr Opin Psychiatry. 2019;28:151–158. doi: 10.1016/j.copsyc.2018.12.005 30684917

[55] Goleman D, Davidson RJ. Altered traits: Science reveals how meditation changes your mind, brain, and body. New York (NY): Penguin; 2017.

[56] Kirby JN, Doty JR, Petrocchi N, Gilbert P. The current and future role of heart rate variability for assessing and training compassion. Front Public Health. 2017;5:40. doi: 10.3389/fpubh.2017.00040 28337432 PMC5340770

[57] Leaviss J, Uttley L. Psychotherapeutic benefits of compassion-focused therapy: An early systematic review. Psychol Med. 2015;45:927–945. doi: 10.1017/S0033291714002141 25215860 PMC4413786

[58] Gilbert P. The origins and nature of compassion focused therapy. Br J Clin Psychol. 2014;53(1):6–41. doi: 10.1111/bjc.12043 24588760

[59] Kirby J, Gilbert P. The emergence of the compassion focused therapies. In: Gilbert P, editor Compassion: Concepts, research and applications. London: Routledge; 2017.p. 258–285

[60] Gilbert P. Compassion focused therapy: Distinctive features. London: Routledge; 2010.

[61] Gilbert P. Compassion: From its evolution to a psychotherapy. Front Psychol. 2020;11:3123. doi: 10.3389/fpsyg.2020.586161 33362650 PMC7762265

[62] Craig C, Hiskey S, Spector A. Compassion focused therapy: a systematic review of its effectiveness and acceptability in clinical populations. Expert Rev Neurother. 2020;20(4):385–400. doi: 10.1080/14737175.2020.1746184 32196399

[63] Kirby JN, Tellegen CL, Steindl SR. A meta-analysis of compassion-based interventions: Current state of knowledge and future directions. Behav Ther. 2017;48: 778–792. doi: 10.1016/j.beth.2017.06.003 29029675

[64] Porges SW. The polyvagal perspective. Biol Psychol. 2007;74(2):116–143. doi: 10.1016/j.biopsycho.2006.06.009 17049418 PMC1868418

[65] Irons C, Beaumont E. The compassionate mind workbook: A step-by-step guide to developing your compassionate self. Cave Junction (OR): Robinson; 2017.

[66] Irons C, Heriot‐Maitland C. Compassionate Mind Training: An 8‐week group for the general public. Psychol Psychother: Theory Res Pract. 2020. doi: 10.1111/papt.12320 33222375

[67] Matos M, Duarte C, Duarte J, Pinto-Gouveia J, Petrocchi N, Basran J, et al. Psychological and physiological effects of compassionate mind training: A pilot randomised controlled study. Mindfulness. 2017;8(6):1699–1712. doi: 10.1007/s12671-017-0745-7

[68] Beaumont E, Irons C, Rayner G, Dagnall N. Does Compassion-Focused Therapy training for health care educators and providers increase self-compassion and reduce self-persecution and self-criticism? J Contin Educ Health Prof. 2016;36(1):4–10. doi: 10.1097/CEH.0000000000000023 26954239

[69] Beaumont EA, Bell T, McAndrew SL, Fairhurst HL. The impact of Compassionate Mind Training on qualified health professionals undertaking a Compassion Focused Therapy module. Couns Psychother Res. 2021;00:1–13. doi: 10.1002/capr.12396

[70] Beaumont E, Martin CJH. Heightening levels of compassion towards self and others through use of compassionate mind training. Br J Midwifery. 2016;24(11):777–786. doi: 10.12968/bjom.2016.24.11.777

[71] McEwan K, Minou L, Moore H, Gilbert P. Engaging with distress: Training in the compassionate approach. J Psychiatr Ment Health Nurs. 2020;27(6):718–727. doi: 10.1111/jpm.12630 32187418

[72] Beaumont E, Durkin M, McAndrew S, Martin C. Using compassion focused therapy as an adjunct to trauma-focused CBT for fire service personnel suffering with trauma-related symptoms. Cogn Behav Therap. 2016;9. doi: 10.1017/S1754470X16000209

[73] Beaumont E, Rayner G, Durkin M, Bowling G. The effects of Compassionate Mind Training on student psychotherapists. J Ment Health Train Educ Pract. 2017;12(5):300–312. doi: 10.1108/JMHTEP-06-2016-0030

[74] Matos M, Duarte C, Duarte J, Gilbert P, Pinto-Gouveia J. How one experiences and embodies compassionate mind training influences its effectiveness. Mindfulness. 2018;9(4):1224–1235. doi: 10.1007/s12671-017-0864-1

[75] Park G, Thayer JF. From the heart to the mind: cardiac vagal tone modulates top-down and bottom-up visual perception and attention to emotional stimuli. Front Psychol. 2014;5:278. doi: 10.3389/fpsyg.2014.00278 24817853 PMC4013470

[76] Petrocchi N, Ottaviani C, Couyoumdjian A. Compassion at the mirror: Exposure to a mirror increases the efficacy of a self-compassion manipulation in enhancing soothing positive affect and heart rate variability. J Posit Psychol. 2016;1–12. doi: 10.1080/17439760.2014.994223 26640507 PMC4666321

[77] Stellar JE, Cohen A, Oveis C, Keltner D. Affective and physiological responses to the suffering of others: Compassion and vagal activity. J Pers Soc Psychol. 2015;108(4):572–585. doi: 10.1037/pspi0000010 25621856

[78] Svendsen JL, Osnes B, Binder PE, Dundas I, Visted E. Nordby H, et al. Trait self-compassion reflects emotional flexibility through an association with high vagally mediated heart rate variability. Mindfulness. 2016;7(5):1103–1113. doi: 10.1007/s12671-016-0549-1 27642372 PMC5010618

[79] Sommers-Spijkerman MPJ, Trompetter HR, Schreurs KMG, Bohlmeijer ET. Compassion-focused therapy as guided self-help for enhancing public mental health: A randomized controlled trial. J Consult Clin Psychol. 2018;86(2):101–115. doi: 10.1037/ccp0000268 29265836

[80] Welford M, Langmead K. Compassion-based initiatives in educational settings. Educ Child Psychol. 2015;32(1):71–80. Available from: https://www.researchgate.net/profile/Mary-Welford-2/publication/320584562_Compassion-based_initiatives_in_educational_settings/links/59eefef64585152de64db43a/Compassion-based-initiatives-in-educational-settings.pdf

[81] Monteiro R, Ucha L, Alvarez T, Milagre C, Neves MJ, Silva M, et al. Estratégia nacional de educação para a cidadania [Internet]. Lisbon: XXI Governo Constitucional: 2017 Sept [cited 2021 Sept]. Available from: https://www.dge.mec.pt/sites/default/files/Projetos_Curriculares/Aprendizagens_Essenciais/estrategia_cidadania.pdf

[82] Direção Geral de Educação and DGS Direção Geral da Saúde. Referencial de Educação para a Saúde. Lisbon (Portugal): Direção-Geral da Educação and Direção-Geral da Saúde; 2017. Available from: https://www.dge.mec.pt/sites/default/files/Esaude/referencial_educacao_saude_vf_junho2017.pdf

[83] Hanh, Weare K. Happy teachers change the world. Berkeley (CA): Parallax Press; 2017.

[84] Hwang YS, Bartlett B, Greben M, Hand K. A systematic review of mindfulness interventions for in-service teachers: A tool to enhance teacher wellbeing and performance. Teach Teach Educ. 2017;64:26–42. doi: 10.1016/j.tate.2017.01.015

[85] Zarate K, Maggin DM, Passmore A. Meta‐analysis of mindfulness training on teacher well‐being. Psychol Sch. 2019;56(10):1700–1715. doi: 10.1002/pits.223

[86] Maratos FA, Gilbert T, Gilbert P. Improving well-being in higher education: Adopting a compassionate approach. In: Gibbs P, Jameson J, Elwick A, editors. Values of the university in a time of uncertainty. Cham: Springer; 2019, p. 261–259.

[87] Matos M, Palmeira L, Albuquerque I, Cunha M, Pedroso Lima M, Galhardo A, et al. Building compassionate schools: Pilot study of a Compassionate Mind Training intervention to promote teachers’ well-being. Mindfulness. 2021. 10.1007/s12671-021-01778-3

[88] Duarte J, McEwan K, Barnes C, Gilbert P, Maratos FA. Do therapeutic imagery practices affect physiological and emotional indicators of threat in high self-critics? Psychol. Psychother: Theory Res Pract. 2015;88(3):270–284. doi: 10.1111/papt.12043 25347984

[89] Longe O, Maratos FA, Gilbert P, Evans G, Volker F, Rockliff H, et al. Having a word with yourself: Neural correlates of self-criticism and self-reassurance. NeuroImage. 2010;49(2):1849–1856. doi: 10.1016/j.neuroimage.2009.09.019 19770047

[90] Rockliff H, Karl A, McEwan K, Gilbert J, Matos M, Gilbert P. Effects of intranasal oxytocin on compassion focused imagery. Emotion. 2011;11(6):1388–1396. doi: 10.1037/a0023861 21707149

[91] Maratos FA, Matos M, Albuquerque I, Wood W, Palmeira L, Cunha M, et al. Exploring the international utility of progressing Compassionate Mind Training in school settings: A comparison of implementation effectiveness of the same curricula in the UK and Portugal. Psychol Educ Rev. 2020;44(2):73–82. doi: 10545/625433

[92] Hemming K, Haines TP, Chilton PJ, Girling AJ, Lilford RJ. The stepped wedge cluster randomised trial: rationale, design, analysis, and reporting. BMJ. 2015;350. doi: 10.1136/bmj.h391 25662947

[93] Gilbert P, Catarino F, Duarte C, Matos M, Kolts R, Stubbs J, et al. The development of compassionate engagement and action scales for self and others. J Compassionate Health Care. 2017;4(1):4. doi: 10.1186/s40639-017-0033-3

[94] Schulz K F, Altman D G, Moher D. CONSORT 2010 statement: Updated guidelines for reporting parallel group randomized trials. Journal of Clin Epidem. 2010;63:834–840. doi: 10.1016/j.jclinepi.2010.02.005 20346629

[95] APA Publications and Communications Board Working Group on Journal Article Reporting Standards. Reporting standards for research in psychology: Why do we need them? What might they be? American Psychologist. 2008;63:839–851. 10.1037/0003-066X.63.9 PMC2957094 19086746

[96] Bowen DJ, Kreuter M, Spring B, Cofta-Woerpel L, Linnan L, Weiner D, et al. How we design feasibility studies. Am J Prev Med. 2009;36(5):452–457. doi: 10.1016/j.amepre.2009.02.002 19362699 PMC2859314

[97] Gilbert P, McEwan K, Mitra R, Franks L, Richter A, Rockliff H. Feeling safe and content: A specific affect regulation system? Relationship to depression, anxiety, stress, and self-criticism. J Posit Psychol. 2008;3(3):182–191. doi: 10.1080/17439760801999461

[98] Pinto-Gouveia José; Dinis Alexandra; Matos Marcela. Types of Positive Affect Scale. [Portuguese translation] Unpublished manuscript. 2008.

[99] Diener ED, Emmons RA, Larsen RJ, Griffin S. The satisfaction with life scale. J Pers Assess. 1985;49(1):71–75. doi: 10.1207/s15327752jpa4901_13 16367493

[100] Albuquerque I, Palmeira L, Lima MP, Cunha M, Galhardo A, Matos M. Measuring the satisfaction with professional life of teachers: Psychometric validation in a Portuguese sample. Forthcoming 2021.

[101] Lovibond PF, Lovibond SH. The structure of negative emotional states: Comparison of the Depression Anxiety Stress Scales (DASS) with the Beck Depression and Anxiety Inventories. Behav Res Ther. 1995;33(3):335–343. doi: 10.1016/0005-7967(94)00075-u 7726811

[102] Pais-Ribeiro JL, Honrado A, Leal I. Contribuição para o estudo da adaptação portuguesa das escalas de ansiedade, depressão e stress (EADS) de 21 itens de Lovibond e Lovibond. Psicologia, Saúde & Doenças. 2004;5(2):229–239. doi: 10216/6910/2/81876.pdf

[103] Antony MM, Bieling PJ, Cox BJ, Enns MW, Swinson RP. Psychometric properties of the 42-item and 21-item versions of the Depression Anxiety Stress Scales in clinical groups and a community sample. Psychol Assess. 1998;10(2): 176–181. doi: 10.1037/1040-3590.10.2.176

[104] Armon G, Shirom A, Melamed S. The Big Five personality factors as predictors of changes across time in burnout and its facets. J Pers. 2012;80(2):403–427. doi: 10.1111/j.1467-6494.2011.00731.x 21449937

[105] Gomes AR. Medida de “Burnout” de Shirom-Melamed (MBSM). Unpublished technical report 2012. Escola de Psicologia, Universidade do Minho.

[106] Baganha C, Gomes AR, Esteves A. Stresse ocupacional, avaliação cognitiva, *burnout* e comprometimento laboral na aviação civil. Psicologia, Saúde & Doenças, 17;2:164–179. 10.15309/16psd170212. 2016

[107] Matos M, Pinto-Gouveia J, Duarte C, Duarte J. Compassionate Engagement and Action Scales for self and others. [Portuguese translation] Unpublished manuscript. 2015.

[108] Steindl SR, Tellegen CL, Filus A, Seppala E, Doty JR, Kirby JN. The Compassion Motivation and Action Scales: a self-report measure of compassionate and self-compassionate behaviours. Aust Psychol. 2021. doi: 10.1080/00050067.2021.1893110

[109] Matos M, Gonçalves E, Palmeira L, Melo I, Steindl S, & Gomes A. Advancing the Assessment of Compassion: Psychometric Study of the Compassion Motivation and Action Scales in a Portuguese Sample. Curr Psychol. 2021. 10.1007/s12144-021-02311-4

[110] Gilbert P, McEwan K, Matos M, Rivis A. Fears of compassion: Development of three self-report measures. Psychol Psychother: Theory Res Pract. 2011;84:239–255. doi: 10.1348/147608310X526511 22903867

[111] Matos M, Pinto-Gouveia J, Duarte J, Simões D. The Fears of Compassion Scales. [Portuguese translation] Unpublished manuscript. 2016.

[112] Gilbert P, Clarke M, Hempel S, Miles JN, Irons C. Criticizing and reassuring oneself: An exploration of forms, styles and reasons in female students. Brit J of Clin Psych. 2004; 43(Pt 1):31–50. doi: 10.1348/014466504772812959 15005905

[113] Castilho P, Pinto‐Gouveia J, Duarte J. Exploring self‐criticism: Confirmatory factor analysis of the FSCRS in clinical and non-clinical samples. Clin Psychol Psychother. 2015; 22(2):153–164. doi: 10.1002/cpp.1881 24307461

[114] Halamová J, Kanovský M, Kupeli N, Gilbert P, Troop N, Zuroff D, et al. The factor structure of the forms of self-criticising, attacking & self-reassuring scale in thirteen distinct populations. J Psychopathol Behav Assess. 2018;40(4):736–751. doi: 10.1007/s10862-018-9686-2 30459486 PMC6223807

[115] Albuquerque I, Matos M, Galhardo A, Cunha M, Palmeira L, Lima M, et al. The Emotional Climate in Organizations Scales: Psychometric properties and factor structure. Unpublished manuscript. 2021.

[116] Gilbert P. The compassionate mind: A new approach to facing the challenges of life. London: Constable Robinson; 2009.

[117] Tarvainen MP, Niskanen JP, Lipponen JA, Ranta-Aho PO, Karjalainen PA. Kubios HRV—heart rate variability analysis software. Comput Methods Programs Biomed. 2014;113(1):210–220. doi: 10.1016/j.cmpb.2013.07.024 24054542

[118] Malik M. Heart rate variability. Standards of measurement, physiological interpretation, and clinical use. Task Force of the European Society of Cardiology and the North American Society of Pacing and Electrophysiology. Eur Heart J .1996;17:354–381. 8737210

[119] Kline RB. Principles and practice of structural equation modelling, 2nd ed. New York (NY): Guilford Press. 2005

[120] Field A. Discovering statistics using IBM SPSS statistics. Thousand Oaks (CA): Sage Publications; 2013.

[121] Tabachnick B, Fidell L. Using multivariate statistics. London: Pearson Education, Inc; 2007.

[122] Neff KD. The development and validation of a scale to measure self-compassion. Self and Identity. 2003;2(3):223–250. doi: 10.1080/15298860309027 26979311

[123] Mather M, Thayer J. How heart rate variability affects emotion regulation brain networks. Curr Opin Behav Sci. 2018;19:98–104. doi: 10.1016/j.cobeha.2017.12.017 29333483 PMC5761738

[124] Elovainio M, Kivimäki M, Puttonen S, Lindholm H, Pohjonen T, Sinervo T. Organizational injustice and impaired cardiovascular regulation among female employees. Occup Environ Med. 2006;63(2):41–144. org.jerome.stjohns.edu/10.1136/oem.2005.019737 10.1136/oem.2005.019737 PMC2078070 16421394

[125] Herr RM, Bosch JA, van Vianen AEM, Jarczok MN, Thayer JF, Li J. Organizational justice is related to heart rate variability in white-collar workers, but not in blue-collar workers—Findings from a cross-sectional study. Ann Behav Med. 2015;49(3):434–448. doi: 10.1007/s12160-014-9669-9 25472852

[126] Fredrickson BL, Joiner T. Positive emotions trigger upward spirals toward emotional well-being. Psychol Sci. 2002;13(2):172. doi: 10.1111/1467-9280.00431 11934003

[127] Werner AM, Tibubos AN, Rohrmann S, Reiss N. The clinical trait self-criticism and its relation to psychopathology: A systematic review—Update. J Affect Disord. 2019;246:530–547. doi: 10.1016/j.jad.2018.12.069 30599378

[128] Pinto-Gouveia J, Castilho P, Matos M, Xavier A. Centrality of shame memories and psychopathology: The mediation effect of self-criticism. Clin Psychol (New York). 2013;20:323–334. doi: 10.1111/cpsp.12044

[129] Cavalcanti LG, Steindl SR, Matos M, Boschen MJ. Fears of compassion magnify the effects of rumination and worry on the relationship between self-criticism and depression. Curr Psychol. 2021. 1–15. D. oi: doi: 10.1007/s12144-021-01510-3

[130] OFSTED [Internet]. Manchester: OFSTED; 2019. Teacher well-being at work in schools and further education providers; [cited 2021 September 9]. Available from: https://assets.publishing.service.gov.uk/government/uploads/system/uploads/attachment_data/file/819314/Teacher_well-being_report_110719F.pdf

[131] Mohr DC, Spring B, Freedland KE, Beckner V, Arean P, Hollon SD, et al. The selection and design of control conditions for randomized controlled trials of psychological interventions. Psychother Psychosom. 2009;78:275–284. doi: 10.1159/000228248 19602916

[132] Cuijpers P, Cristea IA, Karyotaki E, Reijnders M, Huibers MJH. How effective are cognitive behavior therapies for major depression and anxiety disorders? A meta-analytic update of the evidence. World Psychiatry. 2016;15:245–258. doi: 10.1002/wps.20346 27717254 PMC5032489

[133] Kazdin AE. Treatment as usual and routine care in research and clinical practice. Clin Psychol Rev. 2015;42:168–178. doi: 10.1016/j.cpr.2015.08.006 26431668

[134] Matos M, Duarte C, Duarte J, Pinto-Gouveia J, Petrocchi N, Gilbert P. Cultivating the compassionate self: An exploration of the mechanisms of change in compassionate mind training. Mindfulness. 2021. 10.1007/s12671-021-01717-2

